# Mesoionic tetrazolium-5-aminides: Synthesis, molecular and crystal structures, UV–vis spectra, and DFT calculations

**DOI:** 10.3762/bjoc.17.34

**Published:** 2021-02-08

**Authors:** Vladislav A Budevich, Sergei V Voitekhovich, Alexander V Zuraev, Vadim E Matulis, Vitaly E Matulis, Alexander S Lyakhov, Ludmila S Ivashkevich, Oleg A Ivashkevich

**Affiliations:** 1 Laboratory for chemistry of condensed systems, Research Institute for Physical Chemical Problems of Belarusian State University, Leningradskaya 14, 220006 Minsk, Republic of Belarus; 2 Inorganic chemistry department, Faculty of Chemistry, Belarusian State University, Nezalezhnastsi avenue 4, 220050 Minsk, Republic of Belarus

**Keywords:** aminotetrazoles, DFT, mesoionic compounds, UV–vis spectra, X-ray analysis

## Abstract

Tetrazolium-5-aminides have been prepared by the *tert*-butylation of 5-aminotetrazole and its *N*-methyl derivatives by the *t-*BuOH/HClO_4_ system followed by the treatment of the tetrazolium salts by alkali. The mesoionic compounds have been found to show a higher reactivity of the exocyclic N atom in comparison with 5-aminotetrazoles. The compounds reacted with 1,2-dibromoethane and 5-(methylsulfonyl)-1-phenyl-1*H*-tetrazole with substitution of bromine and methylsulfonyl groups giving the corresponding tetrazolium salts or conjugate aminides. The obtained mesoionic tetrazoles have been characterized by elemental analysis, FTIR, NMR, and UV–vis spectroscopy, TGA/DSC analysis and for 1,3-di-*tert*-butyltetrazolium-5-aminide, its *N*,*N*’-ethylene-bridged bis-derivative and (1,3-di-*tert*-butyl-1*H-*tetrazol-3-ium-5-yl)(1-phenyl-1*H*-tetrazol-5-yl)amide by single crystal X-ray analysis. The structural and spectral features of the tetrazolium-5-aminides are discussed by using quantum-chemical calculations.

## Introduction

**Figure 1:**
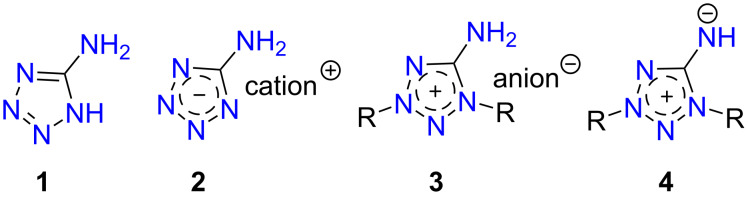
5-Aminotetrazole derivatives.

5-Aminotetrazoles are one of the most available and valuable tetrazole derivatives. So, due to the thermal stability and high nitrogen content the parent 5-aminotetrazole (**1**, Figure 1) is of practical interest as a gas-generator and blowing agent [1–2]. Moreover, it is a useful building block in organic synthesis, including various multicomponent reactions opening the way to diverse fused heterocycles [3]. Salts with anionic tetrazole, i.e., aminotetrazolates **2**, and cationic ones, i.e., aminotetrazolium salts **3**, are attractive as environmentally friendly pyrotechnics [4], insensitive high-energy materials [5–7], and promising energetic ionic liquids [8–10].

The most interesting and less examined 5-aminotetrazole derivatives are tetrazolium-5-aminides, which can be depicted as **4** by analogy with other mesoionic tetrazoles [11–12]. The first representatives of tetrazole-5-aminides were reported in the 1950s [13–14], whereas next publications were appeared only five decades later, being devoted to the synthesis and photochemistry of 1,3-diaryltetrazolium-5-aminides [15–21]. NMR studies of a few tetrazole-5-aminides were carried out as a part of studies of mesoionic compounds [22–24]. Earlier, we reported the facile preparation of 1,3-di-*tert*-butyltetrazolium-5-aminide [25], which was later used for the synthesis of the first tetrazolium halocuprate [26] and also shown to react with bromine-containing alkylation reagents [27]. Very recently, this aminide was found to be a suitable ligand for manganese complexes [28] and used as the agent for the preparation of salts with high energy density [29]. Also, it is worth noting that today only a few examples of mesoionic tetrazole aminide X-ray structures are known [17,28].

Thus, the information on tetrazole-5-aminides is very limited and fragmentary. In the present work, we try to fill this gap by carrying out experimental (synthesis, X-ray, UV–vis) and theoretical density functional theory (DFT) studies of some selected 1,3-dialkyltetrazolium-5-aminides.

## Results and Discussion

### Synthesis and chemical transformations

**Scheme 1:**
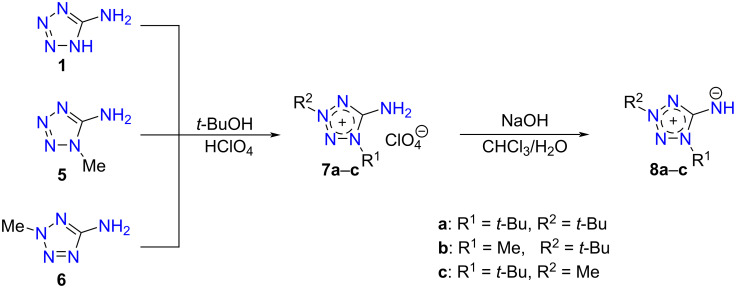
Synthesis of tetrazolium-5-aminides.

Tetrazolium-5-aminides can be prepared by three main approaches: a) by the deprotonation of 5-aminotetrazolium salts, b) by the photochemical transformation of 5-azidotetrazolium salts, and c) by the functionalization of other aminides. The first route is preferable due to the synthetic availability of aminotetrazolium salts [25]. Therefore, here we synthesized 1,3-disubstituted 5-aminotetrazolium perchlorates **7** by the quaternization of commercial 5-aminotetrazole (**1**) and its *N*-methylated derivatives **5** and **6** as shown in Scheme 1. The quaternization proceeded regioselectively using the *t-*BuOH/HClO_4_ system [25]. Further, the salts **7** were treated with sodium hydroxide in the biphasic system water/chloroform giving aminides **8**, which were extracted from the reaction mixtures by chloroform.

The obtained aminides **8** are yellow solids and soluble in various organic solvents, such as alcohols, chloroform, dichloromethane, hexane, acetonitrile, toluene, and THF. They are also soluble in water. Remarkably, the solutions in organic solutions are yellow colored, whereas aqueous solutions are colorless. The UV–vis spectra of **8a** were found to show solvatochromism, that is discussed in more detail in the theoretical section. The ^13^C NMR shift of the C^5^ endocyclic atom for aminides **8** is observed at 162.1–163.1 ppm in DMSO-*d*_6_. For the parent 5-aminotetrazolium salts, this chemical shift is found at 156.5–158.3 ppm in the same solvent. The TGA/DSC data show thermal stability of the tetrazolium-5-aminides (see Supporting Information File 1 for more details).

**Scheme 2:**
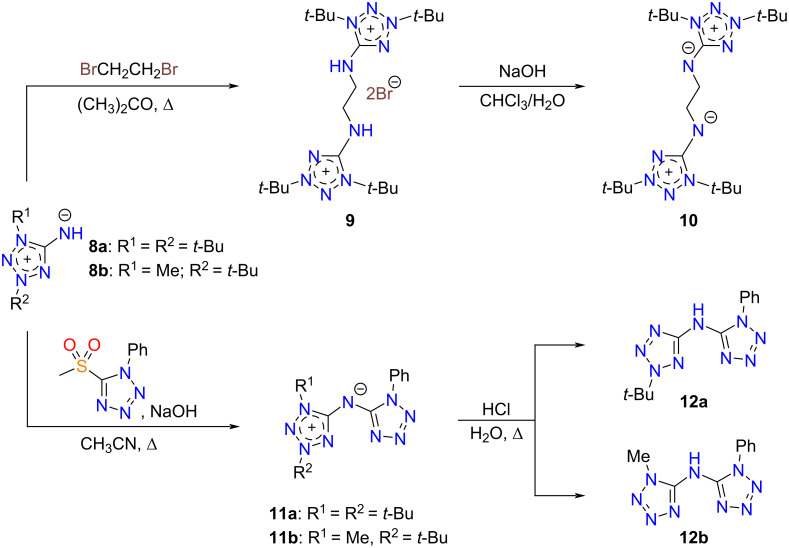
N-Functionalizations of 1,3-disubstituted tetrazolium-5-aminides **8a**,**b**.

Some nucleophilic displacement reactions were carried out in order to show the higher reactivity of the aminides in comparison to the parent aminotetrazoles. The high nucleophilicity of the imine group in tetrazolium-5-aminide allows to displace halo- and methylsulfonyl groups, whereas 5-aminotetrazoles do not react under analogous conditions. So, we prepared the bistetrazolium salt **9** by the alkylation of aminide **8a** with 1,2-dibromoethane (Scheme 2). The obtained salt **9** was subjected to deprotonation to give the bistetrazolium-5-aminide **10**. The reactions of aminides **8a** and **8b** with 5-methylsulfonyl-1-phenyltetrazole in the presence of sodium hydroxide in boiling acetonitrile yielded compounds **11a** and **11b**, respectively.

Taking into account the possibility of removing the *tert*-butyl group in tetrazolium salts under acidic conditions [30], *N*-*tert*-butyltetrazolium-5-aminides are of special interest as agents for the introduction of tetrazol-5-ylamino groups into various substrates having suitable leaving groups. Therefore, we carried out the de-*tert*-butylation of compounds **11a** and **11b** under the action of hydrochloric acid. Indeed, the de-*tert*-butylation was found to be regioselective leading to the 2-*tert*-butyltetrazole (**12a**) and 1-methyltetrazole (**12b**) derivatives, respectively (Scheme 2). The selectivity of the reaction was confirmed by a ^13^C NMR shift comparison for the C^5^ endocyclic atoms of tetrazoles **12a** and **12b**: 161.1 and 151.9 ppm for compound **12a** and 153.2 and 151.1 ppm for compound **12b**. It is known that the ^13^C NMR shifts for the endocyclic C^5^ atom in 2,5-disubstituted tetrazoles is located downfield (162–167 ppm and 151.9 ppm for 2-methyl-5*H*-tetrazole) from the corresponding signal of the 1,5-regioisomers (152–156 and 143.4 ppm for 1-methyl-5*H*-tetrazole) [31]. The de-*tert*-butylation selectivity observed for compound **11b** can be explained by the higher stability of the *t*-Bu cation versus the Me cation.

### Crystal structures

The mesoionic compounds **8a**, **10**, **11a**, and salt **9** were characterized by single crystal X-ray analysis. For all compounds, data collection was performed at a temperature of 100 K and the main crystal data and structure refinement details are given in Table 1.

**Table 1:** Single crystal X-ray data and structure refinement details for compounds **8a**, **10, 11a**, and **9**.

	**8a**	**10**	**11a**	**9**

empirical formula	C_9_H_19_N_5_	C_20_H_40_N_10_	C_16_H_23_N_9_	C_20_H_42_Br_2_N_10_
formula weight	197.29	420.62	341.43	582.45
temperature (K)	100(2)	100(2)	100(2)	100(2)
crystal system	monoclinic	monoclinic	monoclinic	trigonal
space group	*P*2_1_/*c*	*P*2_1_/*c*	*P*2_1_/*n*	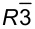
*a* (Å)	5.94661(7)	8.23030(10)	9.04560(10)	36.8562(4)
*b* (Å)	16.2954(2)	10.4082(2)	9.99730(10)	36.8562(4)
*c* (Å)	12.04281(15)	14.7565(2)	20.0685(3)	12.05390(10)
α (°)	90	90	90	90
β (°)	100.2696(6)	94.5711(7)	100.7743(4)	90
γ (°)	90	90	90	120
*V* (Å^3^)	1148.28(2)	1260.06(3)	1782.83(4)	14180.1(3)
*Z*	4	2	4	18
*d*_c_ (g cm^–3^)	1.141	1.109	1.272	1.228
μ (mm^–1^)	0.074	0.072	0.084	2.596
crystal size (mm)	0.56 × 0.31 × 0.22	0.58 × 0.50 × 0.49	0.40 × 0.28 × 0.18	0.50 × 0.10 × 0.09
refl. collected	32431	29074	54434	108400
refl. independ.	4411	3863	8700	9633
restraints	0	0	0	0
parameters	203	196	295	301
GOOF on *F*^2^	1.029	1.072	1.063	1.039
R1/wR2 [I> 2σ(I)]	0.0335/0.0919	0.0371/0.0982	0.0384/0.1027	0.0383/0.0796
R1/wR2 [all data]	0.0361/0.0953	0.0392/0.0998	0.0461/0.1095	0.0639/0.0871
# CCDC	2035296	2035297	2035298	2035299

**Figure 2:**
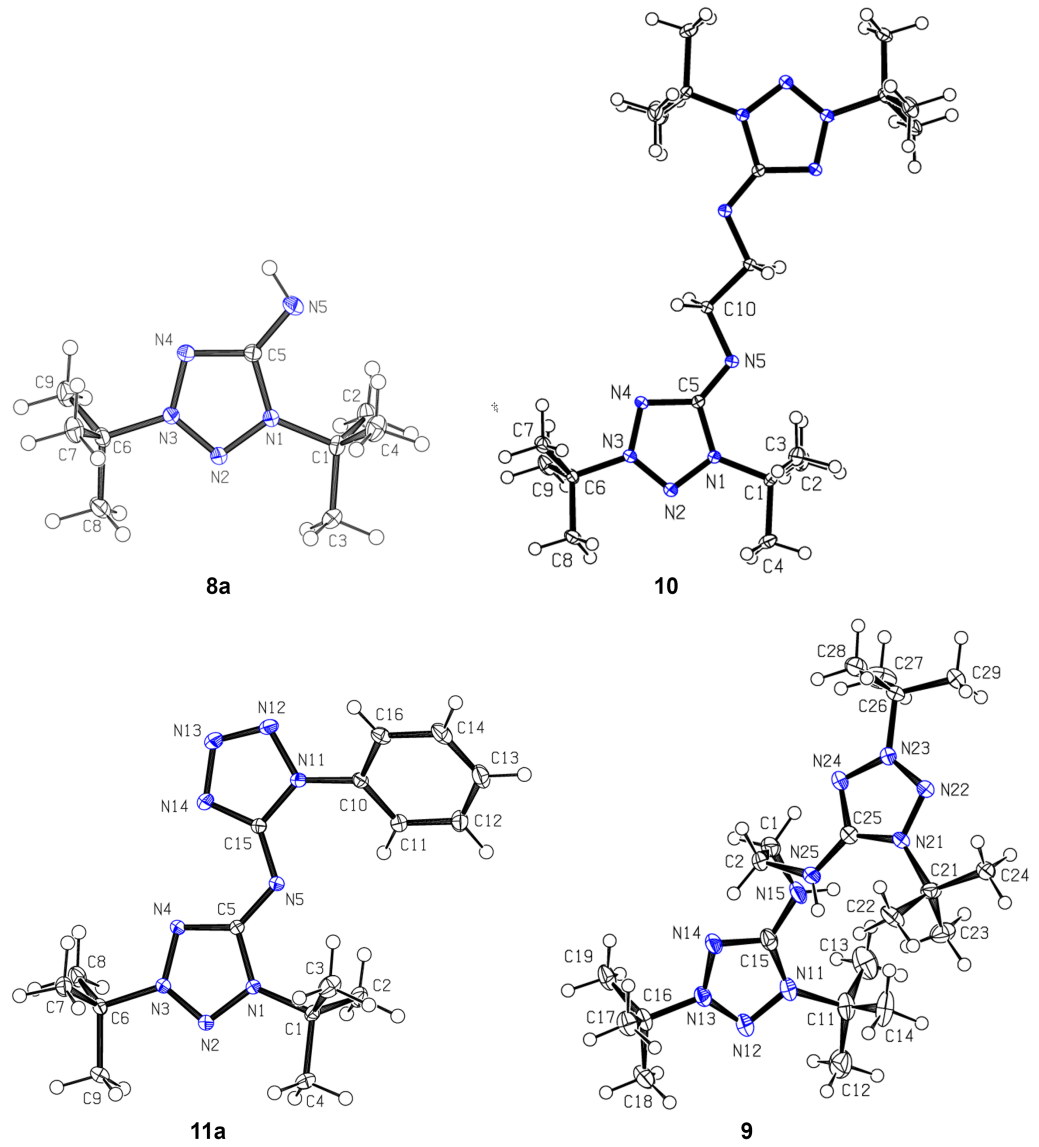
Molecules of compounds **8a**, **10**, **11a**, and the bistetrazolium cation **9**, with displacement ellipsoids drawn at the 50% probability level. The hydrogen atoms are shown as spheres of arbitrary radii. The atom numbering is done for the asymmetric unit.

The mesoionic compounds **8a**, **10**, and **11a** all are monoclinic, with the space groups *P*2_1_/*c* for **8a** and **10**, and *P*2_1_/*n* for **11a**, respectively. The asymmetric units of compounds **8a** and **11a** contain one molecule, whereas the unit of **10** includes a half molecule. In contrast to **8a** and **11a**, the molecule of **10** shows *C*_i_ symmetry, with the inversion center lying in the middle of the bond C10–C10^a^ [symmetry code: (a) –*x* + 2, −*y*, −*z* + 1]. The structures of the molecules are shown in Figure 2.

The bond lengths in the tetrazole cycles and exocyclic C–N bonds in compounds **8a**, **10**, and **11a** are given in Table 2. In these compounds, the shortest bonds are the endocyclic N2–N3 and the exocyclic C5–N5 bonds, being close to double bonds in lengths. However, note that the C5–N5 bond in compound **11a** is somewhat longer compared to those in **8a** and **10**. This fact can be attributed to some electron density shift in **11a** from this bond to the neighboring N5–C15 bond, which is exocyclic in the other tetrazole ring N11/C15 and shows the length of 1.3497(7) Å. The longest endocyclic bonds are N1–C5 and N4–C5, lying in the ranges 1.3892(7)–1.4101(9) and 1.3551(7)–1.3784(9) Å, respectively. The remaining N1–N2 and N3–N4 bonds show close lengths, ranging from 1.3333(7) to 1.3419(6) Å.

**Table 2:** The lengths of the tetrazole ring and exocyclic C–N bonds (Å) in compounds **8a**, **10**, and **11a**, and in the 1,3-di-*tert*-butyltetrazolium-5-aminide ligand in manganese complexes [29].

entry	bond	**8a**	**10**	**11a**	reference [29]

1	N1–C5	1.4034(6)	1.4101(9)	1.3892(7)	1.386(3), 1.390(3)
2	N1–N2	1.3419(6)	1.3396(8)	1.3333(7)	1.352(3), 1.346(3)
3	N2–N3	1.2937(6)	1.2944(8)	1.2935(7)	1.295(3), 1.291(3)
4	N3–N4	1.3350(6)	1.3397(8)	1.3411(7)	1.331(3), 1.337(3)
5	N4–C5	1.3784(7)	1.3842(9)	1.3551(7)	1.355(3), 1.358(3)
6	C5–N5	1.2986(7)	1.2869(9)	1.3241(7)	1.316(3), 1.315(3)

In Table 2, we also included the bond lengths of the 1,3-di-*tert*-butyltetrazolium-5-aminide ligand in a manganese complex [29], being the only structurally characterized complex with a neutral 1,3-dialkyltetrazolium-5-aminide. Therefore, it is of interest to compare the structural data for this compound with those obtained for compound **8a** to find the influence of the complexation on the ligand structure. As can be seen, the bond lengths of the free 1,3-di-*tert*-butyltetrazolium-5-aminide **8a** and the ligand in the manganese complex are rather close. Nevertheless, the following structural differences attract attention. In the complex, the endocyclic N1–C5 and N4–C5 bonds are shorter, but the exocyclic C5–N5 bond is longer compared to aminide **8a**. One can expect that these structural differences are due to the complexation.

It should be noted that the bond lengths of the tetrazole ring N11/C15 in compound **11a** are usual for 1- and 1,5-substituted tetrazoles. The compounds **8a**, **10**, and **11a** show no hydrogen bonds in their crystal structures and only van der Waals interactions take place between the molecules.

The bistetrazolium salt **9** (bromide salt of mesoionic compound **10**) crystallizes in the trigonal space group 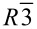, with 18 formula units in the unit cell. The asymmetric unit includes one cation, shown in Figure 2, and two bromide anions. The structure of the cation is close to *C*_2_ symmetry, with an rms deviation of its non-hydrogen atoms from ideal positions of 0.1320 Å. In the salt **9**, the lengths of the tetrazole ring bonds and the exocyclic C–N bond are given in Table 3 together with the data for 1,3-di-alkyltetrazolium-5-aminide salts as described in the literature [25–26,28,32–35]. First of all, it should be mentioned that the formation of the salt from the corresponding mesoionic compound followed by protonation of the endocyclic N atom in all cases presented in Table 3. As can be seen, a good agreement of the bond lengths is observed for each of the six bonds presented in Table 3, and the methyl- and *tert*-butyl derivatives show no differences. On the other hand, a comparison of the data in Table 2 and Table 3 reveals the following structural differences of the salts and the mesoionic compounds: a) the bonds 1 and 5 in the salts are shorter in comparison with the mesoionic compounds and b) the bonds 6 (exocyclic C–N) are somewhat longer in the salts. Hence, when the salt is formed the same trends in structural changes in the mesoions are observed as under their complexation.

**Table 3:** The bond lengths of the tetrazole ring and exocyclic C–N bonds (Å) in salt **9** and the corresponding literature data for 1,3-di-alkyltetrazolium-5-aminide salts^a,b^.

bond numbering	bond #	salt **9**	*R* = Me [32–35]	*R* = *t*-Bu [25–26,28]

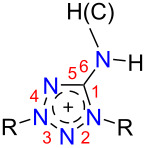	1	1.366(3), 1.359(3)	1.354–1.360	1.365–1.370
	2	1.344(2), 1.347(2)	1.332–1.340	1.340–1.342
	3	1.280(2), 1.286(2)	1.287–1.295	1.284–1.286
	4	1.341(2), 1.336(2)	1.337–1.346	1.334–1.347
	5	1.332(3), 1.333(3)	1.333–1.344	1.332–1.338
	6	1.342(3), 1.334(2)	1.322–1.335	1.322–1.335

^a^Literature data for the corresponding bond are given as a range including bond lengths in different salts. ^b^Bond sequence 1–6 corresponds to that in Table 2.

In salt **9**, the bromide ions are held in the crystal structure by hydrogen bonds N15–H15···Br1^b^ [*D*···*A =* 3.2774(17) Å, *D*–H···*A* = 142°; symmetry code: (b) –*x* + *y* + ^1^/_3_, −x + ^2^/_3_, *z* + ^2^/_3_] and N25–H25···Br2 (*D*···*A* = 3.2654(17) Å, *D*–H···*A =* 144°). There are also intramolecular hydrogen bonds of the methylene H atoms C2–H2A···N14 [*D*···*A* = 3.121(3) Å, *D*–H···*A =* 116°].

### Theoretical study of structures, UV–vis spectra and the experimental UV–vis spectra of compound **8a**

**Scheme 3:**
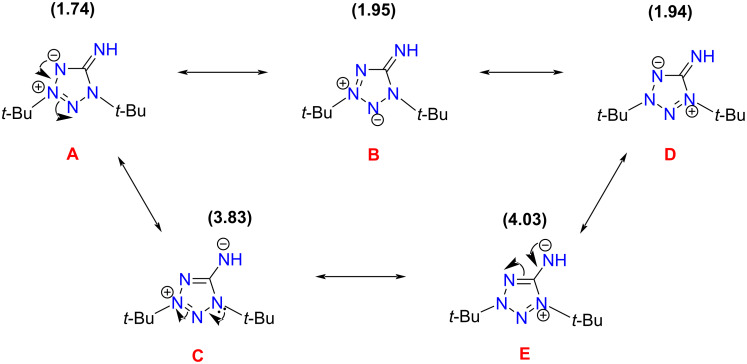
Possible Lewis structures for the molecule of **8a**, with non-Lewis occupancies as % of the total electron density (given in parentheses).

The quantum-chemical study of structure and UV–vis spectra, as well as the experimental study of UV–vis spectra were carried out for compound **8a** being the simplest mesoionic compound among the investigated ones in the present work. The atom numbering used in this section corresponds to that shown in Figure 2. The molecule of **8a** can be represented by several Lewis structures (Scheme 3).

To find the best Lewis structure for the molecule of **8a**, a natural bond orbital (NBO) analysis was applied. In terms of the NBO theory, the "best Lewis structure" is the structure with the lowest non-Lewis occupancy. The NBO search for the molecule of **8a** led to the best Lewis structure **A** (Scheme 3). The high non-Lewis occupancy shows a strong electron delocalization in the molecule of **8a**. To study the nature of the electron delocalization for the structure **A**, an analysis of the interactions between donor Lewis-type NBOs and acceptor non-Lewis NBOs was performed. It showed that the p-type lone pair on the N4 atom is strongly delocalized into vicinal N2–N3* and C5–N5* antibonds, leading to the Lewis structures **B** and **C**, correspondingly (Scheme 3). A similar analysis of donor–acceptor interactions for the obtained structures leads to the Lewis structures **D** and **E**. Note that the structures **B** and **D** with a C5–N5 double bond have only a slightly higher non-Lewis occupancy in comparison with structure **A**, and hence they are also good Lewis structures. Thus, the structures **A**, **B**, and **D** make a greater contribution to the overall structure of **8a** in comparison with structures **C** and **E** having a C5–N5 single bond.

**Figure 3:**
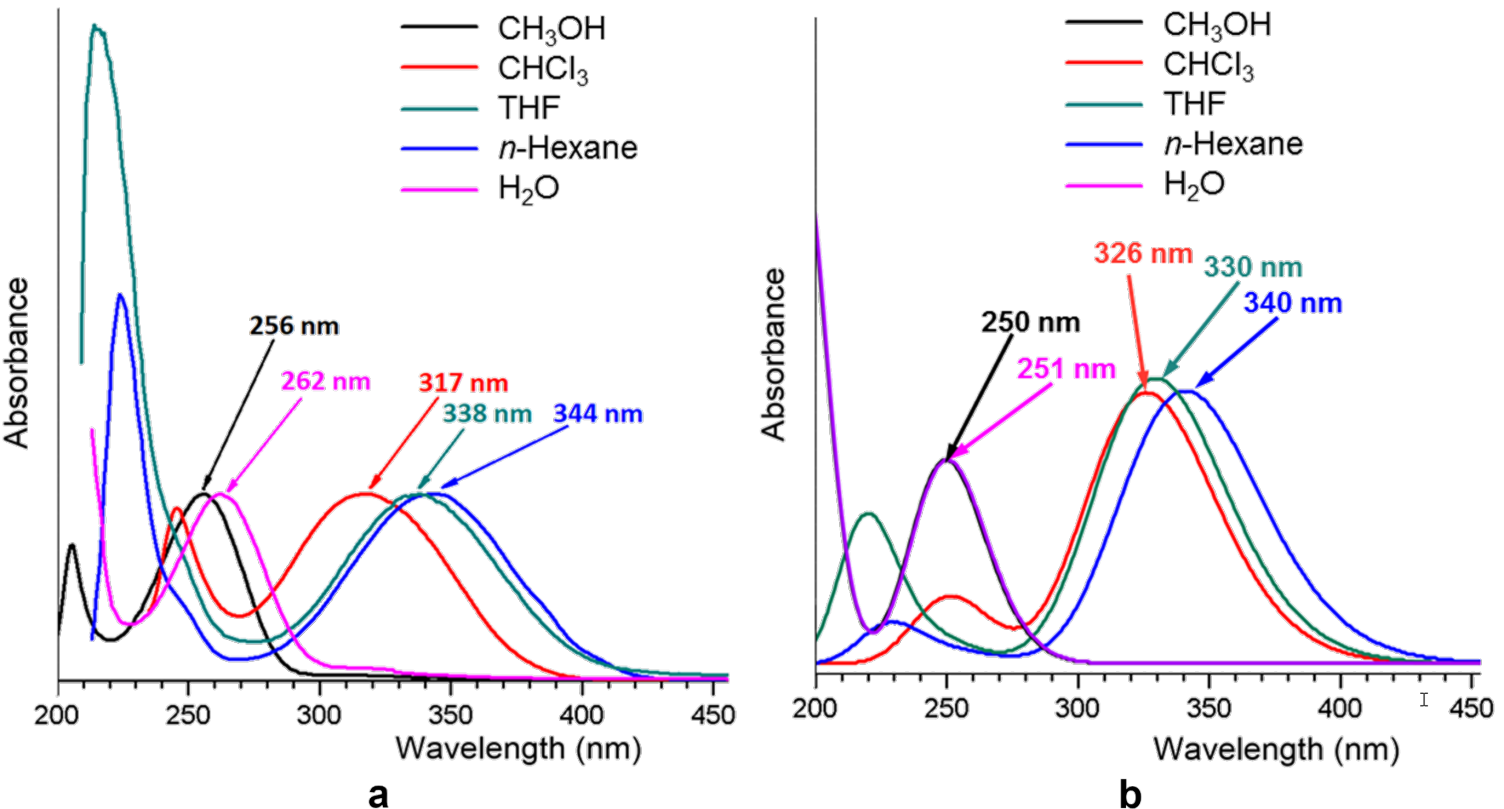
Experimental (a) and TD-tHCTHhyb/6-311+G(2d,p) calculated (b) UV–vis spectra of compound **8a** in different solvents. The model structures of **8a**, shown in Figure 4 were used in the calculations.

**Figure 4:**
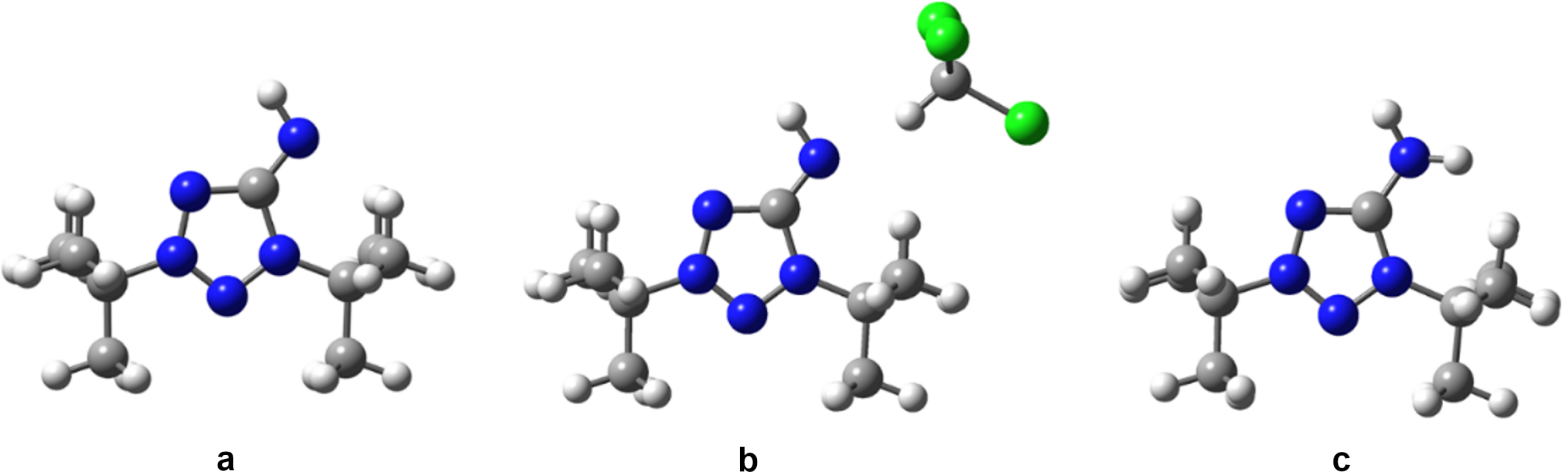
Model structures of **8a** used for the calculations of the UV–vis spectra: a) In *n*-hexane and THF, b) in chloroform, and c) in methanol and water.

**Figure 5:**
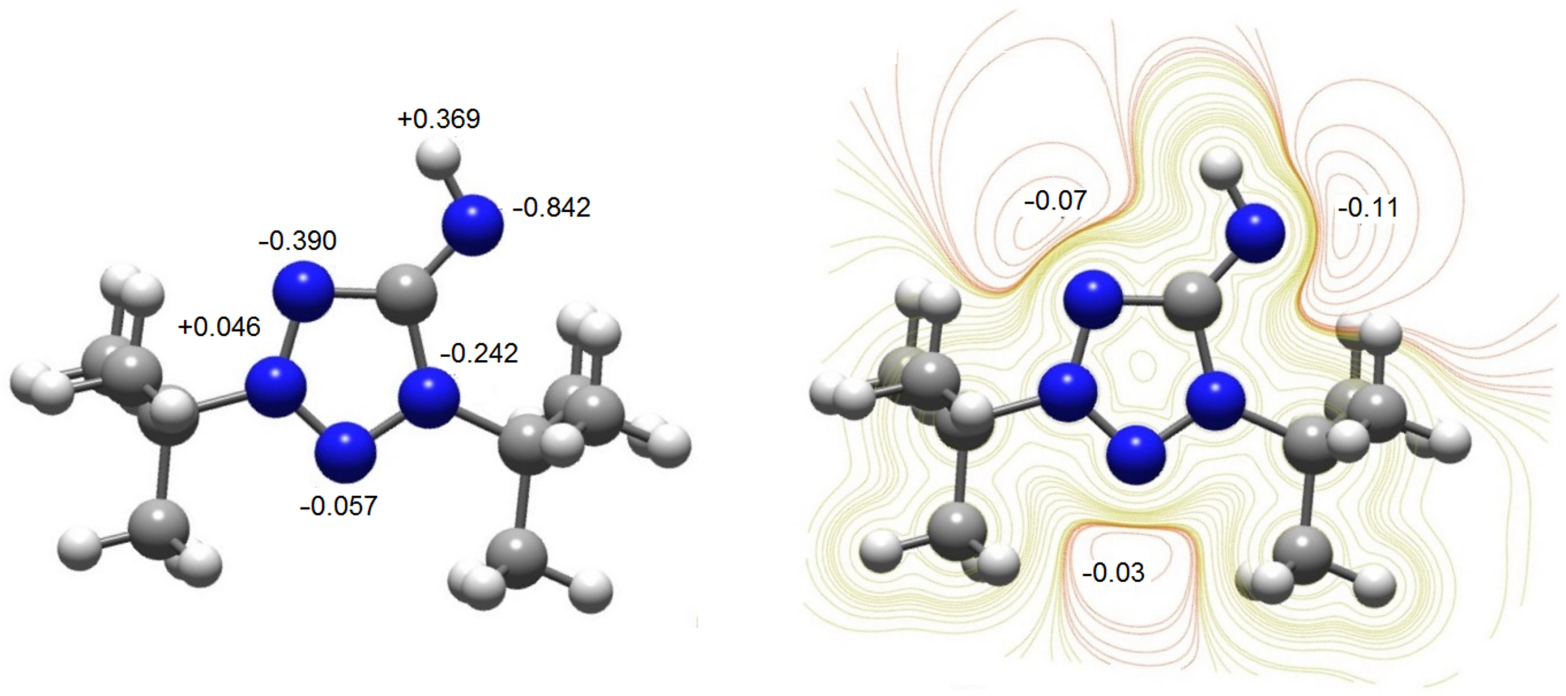
NPA charges (left) and MESP contour map (right) for the molecule of **8a**.

As mentioned earlier, compounds **8a**–**c** show solvatochromism. The experimental UV–vis spectra of compound **8a** in different solvents are presented in Figure 3a. As can be seen, the experimental spectra in *n*-hexane and THF, being similar, differ significantly from those in chloroform, methanol, and water. This difference may be due to the following reasons: in case of chloroform, the solvent can form hydrogen bonds with the nitrogen atoms of the **8a** molecule, whereas for methanol and water solutions, the solvent can act as a proton donor, and as a result, compound **8a** may exist in the 1,3-di-*tert*-butyl-5-aminotetrazolium cationic form. To confirm this assumption, we calculated the UV–vis spectra of **8a** in different solvents for the model structures shown in Figure 4. The model structures in Figure 4b and Figure 4c were built based on the results of our calculations of NPA charges and the molecular electrostatic potential (MESP, Figure 5), showing that the largest negative charge and the deepest minimum of MESP of **8a** are located near the exocyclic atom N5, and hence it is the most preferable protonation site in **8a**.

The TD-tHCTHhyb/6-311+G(2d,p) calculated UV–vis spectra of compound **8a** in *n*-hexane, THF, chloroform, methanol, and water are presented in Figure 3b. As can be seen, for *n*-hexane and THF solutions, the experimental and calculated spectra are in good agreement in the case of the model structure in Figure 4a. However, in the case of a chloroform solution, the calculated spectrum agrees with the experimental one only if the formation of a hydrogen bond between the exocyclic N5 atom and the solvent is taken into account (model structure in Figure 4b). For the methanol and water solutions, the agreement between the calculated and experimental spectra is observed only when the protonation of the N5 atom is taken into account (model structure in Figure 4c). For more details, see Supporting Information File 1.

**Figure 6:**
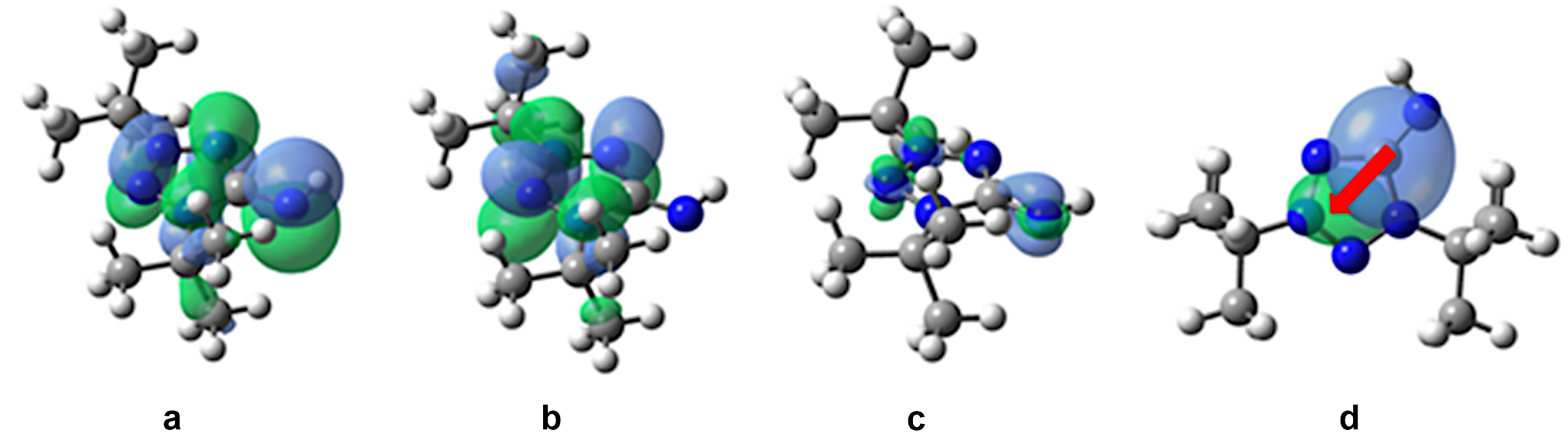
The calculated plots in *n*-hexane of a) HOMO, b) LUMO, c) electron density difference between the S_1_ and S_0_ states, and d) the S_0_→S_1_ CT. Green (blue) regions (for c and d) indicate an increase (decrease) in the electron density upon the electronic transition. The red arrow shows the charge transfer.

**Figure 7:**
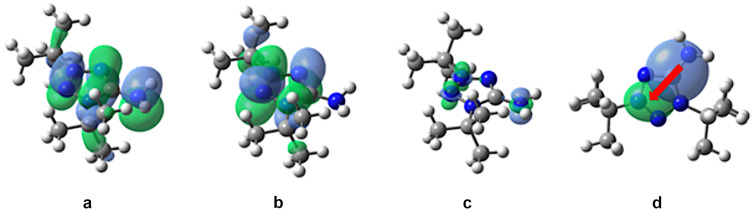
The calculated plots in water of a) HOMO, b) LUMO, c) electron density difference between the S_1_ and S_0_ states, and d) the S_0_→S_1_ CT. Green (blue) regions (for c and d) indicate an increase (decrease) in the electron density upon electronic transition. The red arrow shows the charge transfer.

Charge transfer (CT) during excitation plays a key role in many technological applications because CT states correspond to a light-activated electron–hole separation where the positive and negative charges are distant enough to allow their independent collection. In this case, the charge goes from a donor–acceptor (S_0_) to a donor(+)–acceptor(−) (S_1_). We have studied, how the nature of the solvent can influence the S_0_→S_1_ CT. For this purpose, we calculated plots of the highest occupied molecular orbital (HOMO), the lowest unoccupied molecular orbital (LUMO), the electron density difference between the S_1_ and S_0_ states, as well as the S_0_→S_1_ CT for compound **8a** in the non-polar solvent *n*-hexane (Figure 6) and in a polar water solution (Figure 7).

As it can be seen from the HOMO, LUMO, and electron density difference plots, the S_0_→S_1_ electron excitation corresponds to the π→π* transition in *n*-hexane solution (Figure 6a–c) and to the n→π* transition in water solution (Figure 7a–c). In both cases, the transition is accompanied by a CT from the =N5–H group (donor) to the tetrazole ring (acceptor) (Figure 6d and Figure 7d). For the *n*-hexane and water solutions, the calculated CT distances are 1.5 and 2.0 Å, respectively. The change in the dipole moment, caused by the excitation, is greater in water being 6.1 D vs 4.9 D in *n*-hexane. Moreover, the calculated τ index is much greater in the case of the water solution (0.520 Å in water vs 0.017 Å in *n*-hexane). Note, that a greater positive τ index indicates a stronger charge separation as a result of the electron transition [36]. Hence, in the case of *n*-hexane, the distribution of the positive and negative charge is almost not separated, and the CT is small. However, for the water solution, the distribution of the positive and negative charge is significantly separated due to a strong CT.

Considering that in polar media the S_0_→S_1_ electron excitation corresponds to the n→π* transition, leading to a significant CT from the =N5–H group to the tetrazole ring, we can explain the strong blue shift, observed in methanol and water solution. So, in the ground state, the N5 atom of **8a** has a large negative charge (Figure 5) and strongly interacts with polar solvents. Therefore, polar solvents significantly decrease the ground state energy. When the excited state emerges, the strong CT from the =N5–H group to the tetrazole ring (Figure 6c and d) leads to a decrease in the electronic density on the N5 atom. The solvent molecules do not have time to rearrange in order to stabilize the excited state. This results in a lower ground state energy, but not the excited state. Therefore, the energy of S_0_→S_1_ transition increases, and a blue shift is observed in polar media.

## Conclusion

Mesoionic tetrazolium-5-aminides can be easily prepared by the alkylation of readily available 5-aminotetrazole and its N-alkyl derivatives in a *t-*BuOH/HClO_4_ system followed by the treatment of the tetrazolium salts by alkali. The mesoionic compounds show a higher reactivity of the exocyclic N-atom in comparison with the 5-aminotetrazole ones that can be explained by a unique mesoionic system of the tetrazolium-5-aminides which leads up to a 5-amino-group activation. The compounds react with 1,2-dibromoethane and 5-(methylsulfonyl)-1-phenyl-1*H*-tetrazole and substitute bromine and methylsulfonyl groups giving tetrazolium salts or conjugate aminides. The obtained mesoionic tetrazoles have been characterized by elemental analysis, FTIR, NMR and UV–vis spectroscopy, TGA/DSC analysis, and for 1,3-di-*tert*-butyltetrazolium-5-aminide, its *N*,*N*’-ethylene-bridged bis-derivative and (1,3-di-*tert*-butyl-1*H-*tetrazol-3-ium-5-yl)(1-phenyl-1*H*-tetrazol-5-yl)amide by single crystal X-ray analysis. The structural and spectral features of the tetrazolium-5-aminides were discussed by using quantum-chemical calculations.

## Experimental

CAUTION: The prepared 5-iminotetrazoles and their derivatives are energetic compounds with increased sensitivities against heat. Although we had no problems during synthesis, the use of safety equipment such as leather gloves, face shield, and the use of Teflon spatulas is mandatory.

### General information

Unless otherwise noted, all reagents were obtained from commercial sources and used without further purification. The UV–vis spectra were recorded on a Merertech SP-8001-6C UV–visible spectrophotometer. ^1^H and ^13^C NMR spectra were recorded on a Bruker AVANCE 500 MHz spectrometer. IR spectra were recorded on a Bruker Vertex 70 spectrometer in diamond cell accessory. For Raman spectra registration, an Ocean Optics ID RAMAN READER (785 nm) spectrometer was used.

### Experimental procedures

The experimental procedures are given in Supporting Information File 1.

### Computation details

Calculations of the UV–vis spectra and charge transfer were carried out within density functional theory for ground states and time-dependent density functional theory (TD-DFT) for excited states using the τ-dependent hybrid tHCTHhyb functional [37] with the 6-311+G(2d,p) basis set [38]. The chosen functional allows to predict valence electronic transition energies with high accuracy [39]. However, the tHCTHhyb functional is much less accurate in the calculation of Rydberg electronic transition energies [39]. The geometry of **8a** was fully optimized for the ground state in each solvent – *n*-hexane, THF, chloroform, methanol, water. Solvation effects were considered using the SMD [40] model in terms of Linear Response scheme [41]. The analysis of charge transfer during S_0_→S_1_ transition was carried out by using the Multiwfn software [36]. The NBO analysis and MESP calculations were performed using the B3LYP/6-31G(d) level of theory [42].

### X-ray structure determination

Single crystal X-ray diffraction data of the mesoionic compounds **8a**, **10**, **11a**, and salt **9** were collected on a SMART Apex II diffractometer using graphite monochromatic Mo Kα radiation (λ = 0.71073 Å) at a temperature of 100 K. The structures were solved by direct methods (SIR2014) [43] and refined on *F*^2^ by the full-matrix least squares technique (SHELXL 2014) [44]. The intensities were corrected for absorption. For all compounds, non-hydrogen atoms were refined anisotropically. For compounds **8a**, **10**, and **11a**, all hydrogen atoms were found from the difference Fourier map. For compound **8a**, they were refined isotropically; for compounds **10** and **11a**, the positions of hydrogen atoms were refined with *U*_iso_(H) = 1.5*U*_eq_(C) for the methyl groups and *U*_iso_(H) = 1.2*U*_eq_(C) for others. For salt **9**, the methyl and methylene group H atoms were placed in calculated positions and refined in a “riding model”, with *U*_iso_(H) = 1.5*U*_eq_(C) for the methyl and *U*_iso_(H) = 1.2*U*_eq_(C) for the methylene groups; the hydrogen atoms of N–H groups were found from the difference Fourier map and refined isotropically with *U*_iso_(H) = 1.2*U*_eq_(N). In salt **9**, the structure contains large voids, however, the residual electron density in the voids was difficult to model and therefore, the SQUEEZE routine in PLATON [45] was used to remove the contribution of the electron density in the solvent region from the intensity data and the solvent-free model was employed for the final refinement. The solvent contribution was not included in the reported molecular weight and density. Molecular graphics were performed with the programs ORTEP-3 for Windows [46] and PLATON [47]. CCDC deposition numbers for the compounds are 2035296 (**8a**), 2035297 (**10**), 2035298 (**11a**), and 2035299 (**9**).

## Supporting Information

File 1 Experimental procedures, copies of spectra and calculation results.
