# Supplementary material for: Mesoionic tetrazolium-5-aminides: Synthesis, molecular and crystal structures, UV–vis spectra, and DFT calculations
Source: Beilstein J Org Chem. 2021 Feb 8;17:385–95. doi: 10.3762/bjoc.17.34 (PMC7884879; doi:10.3762/bjoc.17.34)
Supplement: File 1 — Experimental procedures, copies of spectra and calculation results. [file Beilstein_J_Org_Chem-17-385-s001.pdf]

BEILSTEIN JOURNAL OF ORGANIC CHEMISTRY

Supporting Information

for

Mesoionic tetrazolium-5-aminides: Synthesis, molecular and crystal structures, UV–vis spectra, and DFT calculations

Vladislav A. Budevich, Sergei V. Voitekhovich, Alexander V. Zuraev, Vadim E. Matulis, Vitaly E. Matulis, Alexander S. Lyakhov, Ludmila S. Ivashkevich and Oleg A. Ivashkevich

Beilstein J. Org. Chem. **2021,** *17,* 385–395. doi:10.3762/bjoc.17.34

Experimental procedures, copies of spectra and calculation results

License and Terms: This is a supporting information file under the terms of the Creative Commons Attribution License (https://creativecommons.org/licenses/by/4.0). Please note that the reuse, redistribution and reproduction in particular requires that the author(s) and source are credited and that individual graphics may be subject to special legal provisions.
The license is subject to the *Beilstein Journal of Organic Chemistry* terms and conditions: (https://www.beilstein-journals.org/bjoc/terms)

Experimental procedures

General procedure for synthesis of 8a–c. A solution of salt **7a** (**7b** or **7c**) (17 mmol) in chloroform (150 mL) was shaken with a solution of sodium hydroxide (3.41 g, 85 mmol) in water (40 mL), and an intense yellow color immediately appeared. The organic layer was separated, dried with anhydrous sodium sulfate, and concentrated to give the tetrazole-5-aminides **8a** (**8b** or **8c**) as light-yellow crystals. Compound **8a** was crystallized from hexane to give crystals suitable for single crystal X-ray analysis.

1,3-Di-*tert*-butyltetrazolium-5-aminide (8a). Yield 3.05 g (91 %). Mp, ^{1}H and ^{13}C NMR, Anal. Calcd data are given in [25]. UV-Vis, λ_{max} (nm): water – 262; MeOH – 205, 256; $CHCl_3$ – 246, 317; THF – 216, 338; hexane – 224, 344. IR (neat), ṽ: 3327 (w), 3193 (w), 2984 (s), 2941 (s), 2913 (m), 2878 (m), 1610 (s), 1474 (s), 1455 (s), 1420 (m), 1402 (m), 1385 (s), 1369 (s), 1311 (s), 1292 (m), 1175 (s), 1083 (m), 1036 (s), 993 (s), 939 (m), 844 (m), 819 (w), 787 (w), 728 (m), 685 (s), 650 (s), 610 (s), 567 (w), 489 (w), 461 (w), 442 (w), 420 (w) cm^{-1}. Raman, ṽ (rel. int.): 2984 (11), 2928 (21), 2791 (3), 2719 (4), 1620 (14), 1590 (19), 1444 (30), 1403 (54), 1366 (15), 1307 (28), 1288 (5), 1230 (17), 1176 (66), 1149 (16), 1079 (27), 1033 (22), 989 (62), 936 (32), 841 (47), 815 (100), 783 (67), 681 (3), 606 (47), 565 (44), 457 (5), 413 (4), 337 (4), 316 (5), 267 (6) cm^{-1}.

1-Methyl-3-*tert*-butyltetrazolium-5-aminide (8b). Yield 2.31 g (88 %). Mp 48–51 °C. ^{1}H NMR (500.0 MHz, DMSO-d_6), δ: 3.53 (s, 3H, Me), 1.57 (s, 9H, t-Bu) ppm; ^{13}C NMR (125.7 MHz, DMSO-d_6), δ: 163.1 (CN_4), 66.0 ($\underline{C}Me_3$), 31.8 (Me), 28.2 (3Me) ppm. Anal. Calcd for $C_6H_{13}N_5$: C, 46.4; H, 8.4; N, 45.1. Found: C, 46.5; H, 8.2; N, 45.5. IR (neat), ṽ: 3310 (w), 3028 (w), 2991 (m), 2943 (m), 2881 (w), 1824 (w), 1620 (s), 1513 (w), 1462 (s), 1445 (m), 1414 (m), 1373 (s), 1317 (m), 1296 (w), 1223 (s), 1187 (s), 1131 (w), 1100 (w), 1067 (w), 1042 (m), 1006 (w), 988 (m), 943 (w), 934 (w), 843 (s), 736 (s), 675 (s), 589 (m), 570 (m), 513 (w), 460 (w), 438 (w) cm^{-1}.

3-Methyl-1-*tert*-butyltetrazolium-5-aminide (8c). Yield 2.53 g (96 %). Mp 44–46 °C. ^{1}H NMR (500.0 MHz, DMSO-d_6), δ: 4.28 (br, s, 1H, NH), 3.95 (s, 3H, Me), 1.59 (s, 9H, t-Bu) ppm; ^{13}C NMR (125.7 MHz, DMSO-d_6), δ: 162.3 (CN_4), 59.2 (CMe_3), 41.7 (Me), 26.7 (3Me) ppm. Anal. Calcd for $C_6H_{13}N_5$: C, 46.4; H, 8.4; N, 45.1. Found: C, 46.7; H, 8.3; N, 45.4. IR (neat), ṽ: 3372 (m), 3283 (m), 2979 (w), 2920 (m), 2851 (w), 1741 (w), 1654 (m), 1585 (s), 1484 (w), 1456 (m), 1396 (m), 1356 (s), 1228 (s), 1210 (s), 1163 (s), 1112 (m), 1078 (s), 1039 (m), 1012 (m), 933 (w), 851 (m), 807 (m), 791 (m), 737 (m), 658 (s) cm^{-1}.

Bistetrazolium salt 9. To solution of compound **8a** (1.0 g, 5.1 mmol) in acetone (5 mL) 1,2-dibromoethane (0.22 mL, 2.5 mmol) was added and the mixture refluxed for 3 h. The precipitate was filtered, dried, and washed with cold acetone to give salt **9**. Yield 0.36 g (24 %). Mp 187–190 °C. ^{1}H NMR (500.0 MHz, DMSO-d6), δ: 8.04 (s, 2H, NH), 3.68 (s, 4H, $2CH_2$), 1.72 (s, 18H, t-Bu), 1.70 (s, 18H, t-Bu) ppm; ^{13}C NMR (125.7 MHz, DMSO-d6), δ: 156.1 (CN_4), 69.7 ($\underline{C}Me_3$), 64.7 ($\underline{C}Me_3$), 42.0 ($2CH_2$), 28.2 (3Me), 27.1 (3Me) ppm. Anal. Calcd for $C_{20}H_{42}Br_2N_{10}$: C, 41.2; H, 7.3; N, 24.1. Found: C, 41.2; H, 7.0; N, 24.3. IR (neat), ṽ: 3146 (m), 3100 (m), 2986 (m), 2943 (m), 1622 (s), 1542 (w), 1492 (m), 1437 (w), 1409 (w), 1376 (m), 1352 (m), 1312 (w), 1278 (w), 1242 (m), 1234 (m), 1218 (m), 1187 (s), 1167 (m), 1095 (w), 1073 (w), 1046 (w), 1027 (w), 943

(w), 905 (m), 869 (w), 835 (w), 818 (w), 789 (w), 732 (w), 669 (w), 646 (w), 587 (w), 559 (w), 544 (w), 493 (w), 457 (w), 444 (w), 424 (w) cm^{-1}.

Bistetrazolium-5-aminide 10. Compound **9** (0.14 g, 0.33 mmol) was dissolved in chloroform (5 mL), and then a solution of sodium hydroxide (0.132 g, 3.3 mmol) was added. The organic layer was separated, dried with anhydrous sodium sulfate, and concentrated to give **10** as yellow crystals. The obtained compound was recrystallized from toluene to give crystals suitable for single crystal X-ray analysis. Yield 0.10 g (94 %). Mp 192–194 °C. UV-Vis, λ_{max} (nm): MeOH – 206, 263; $CHCl_3$ – 244, 348; THF – 232, 362; hexane – 223, 367. ^1H NMR (500.0 MHz, CD_3CN), δ: 3.32 (s, 4H, $2CH_2$), 1.65 (s, 18H, t-Bu), 1.64 (s, 18H, t-Bu) ppm; ^{13}C NMR (125.7 MHz, CD_3CN), δ: 157.9 (CN_4), 66.1 ($\underline{C}Me_3$), 60.0 ($\underline{C}Me_3$), 49.1 ($2CH_2$), 27.5 (3Me), 26.1 (3Me) ppm. Anal. Calcd for $C_{20}H_{40}N_{10}$: C, 57.1; H, 9.6; N, 33.3. Found: C, 57.4; H, 9.4; N, 33.5. IR (neat), ṽ: 2991 (m), 2979 (m), 2965 (m), 2938 (m), 2915 (m), 2874 (m), 2821 (m), 1625 (s), 1479 (m), 1462 (s), 1437 (w), 1397 (m), 1367 (s), 1352 (m), 1290 (s), 1272 (s), 1253 (s), 1211 (w), 1190 (s), 1173 (s), 1116 (s), 1057 (m), 1035 (m), 990 (m), 937 (m), 855 (m), 800 (w), 719 (m), 670 (w), 648 (m), 598 (s), 564 (w), 491 (m), 430 (w) cm^{-1}. Raman, ṽ (rel. int.): 2979 (17), 2926 (24), 2859 (11), 2815 (17), 2715 (5), 1642 (43), 1449 (59), 1403 (73), 1392 (74), 1368 (28), 1345 (10), 1290 (17), 1274 (26), 1263 (26), 1233 (40), 1170 (51), 1109 (56), 1088 (1088), 1030 (38), 986 (74), 929 (38), 866 (21), 815 (100), 802 (36), 714 (4), 649 (66), 609 (6), 562 (44), 470 (11), 450 (10), 371 (3), 340 (9), 267 (5) cm^{-1}.

(1,3-Di-*tert*-butyl-1*H*-tetrazol-3-ium-5-yl)(1-phenyl-1*H*-tetrazol-5-yl)amide (11a). Compound **8a** (0.43 g, 2.2 mmol) and 5-(methylsulfonyl)-1-phenyl-1*H*-tetrazole (0.45 g, 2.0 mmol) were dissolved in acetonitrile (10 mL). Sodium hydroxide (0.088 g, 2.2 mmol) was added, and the mixture refluxed for 5 h. The precipitate was filtered and discarded. The filtrate was evaporated to dryness, recrystallized from ethyl alcohol to give **11a** as white crystals. After recrystallization from acetonitrile crystals suitable for X-ray analysis were obtained. Yield 0.32 g (46 %). Mp 206–208 °C. ^1H NMR (500.0 MHz, DMSO-d_6), δ: 7.85–7.45 (m, 5H, Ph), 1.70 (s, 9H, t-Bu), 1.69 (s, 9H, t-Bu) ppm; ^{13}C NMR (125.7 MHz, DMSO-d_6), δ: 157.9 (CN_4), 156.4 (CN_4), 135.7 (C^4, Ph), 129.6 ($C^{3,5}$, Ph), 128.4 (C^1, Ph), 123.5 ($C^{2,6}$, Ph), 67.8 ($\underline{C}Me_3$), 62.6 ($\underline{C}Me_3$), 28.3 (3Me), 27.1 (3Me) ppm. Anal. Calcd for $C_{16}H_{23}N_9$: C, 56.3; H, 6.8; N, 37.0. Found: C, 56.3; H, 6.9; N, 37.3. IR (neat), ṽ: 3363 (w), 3061 (w), 2985 (w), 2667 (w), 2924 (m), 2852 (w), 1603 (m), 1568 (s), 1520 (s), 1499 (s), 1475 (m), 1455 (s), 1412 (m), 1403 (m), 1374 (m), 1363 (m), 1344 (m), 1295 (w), 1276 (w), 1264 (w), 1235 (m), 1213 (m), 1178 (s), 1159 (m), 1124 (m), 1093 (s), 1070 (m), 1044 (w), 1035 (w), 1018 (w), 1002 (m), 984 (w), 969 (w), 962 (w), 938 (w), 912 (m), 868 (m), 839 (w), 827 (w), 816 (w), 758 (s), 745 (m), 739 (m), 694 (m), 686 (s) cm^{-1}.

(1-Methyl-3-*tert*-butyl-1*H*-tetrazol-3-ium-5-yl)(1-phenyl-1*H*-tetrazol-5-yl)amide (11b). Compound **8b** (0.16 g, 1.0 mmol) and 5-(methylsulfonyl)-1-phenyl-1*H*-tetrazole (0.22 g, 1.0 mmol) were dissolved in acetonitrile (5 mL). Sodium hydroxide (0.044 g, 1.1 mmol) was added and mixture refluxed for 5 h. The precipitate was filtered and discarded. The filtrate was evaporated to dryness, recrystallized from ethyl alcohol to give **11b** as white crystals. Yield 0.15 g (50 %). Mp 213–215 °C. ^1H NMR (500.0 MHz, DMSO-d_6), δ: 7.99–7.43 (m, 5H, Ph), 3.84 (s, 3H, Me), 1.70 (s, 9H, t-Bu) ppm; ^{13}C NMR (125.7 MHz, DMSO-d_6), δ: 158.7 (CN_4), 156.3 (CN_4), 135.8 (C^4, Ph), 130.0 ($C^{3,5}$,

Ph), 128.1 (C^1, Ph), 122.6 ($C^{2,6}$, Ph), 68.1 (<u>C</u>Me_3), 33.2 (Me), 28.3 (3Me) ppm. Anal. Calcd for $C_{13}H_{17}N_9$: C, 52.2; H, 5.7; N, 42.1. Found: C, 52.3; H, 5.4; N, 42.5. IR (neat), ṽ: 3074 (w), 2987 (w), 1652 (w), 1606 (s), 1587 (s), 1525 (s), 1497 (s), 1457 (s), 1414 (w), 1404 (w), 1393 (s), 1375 (s), 1359 (w), 1327 (m), 1292 (m), 1264 (m), 1228 (m), 1182 (s), 1164 (m), 1134 (s), 1099 (s), 1073 (s), 1037 (m), 1017 (m), 987 (s), 925 (m), 900 (s), 824 (w), 764 (s), 743 (w), 729 (m), 696 (m), 666 (s), 616 (w), 576 (m), 546 (m), 542 (m), 518 (w), 491 (m), 468 (m) cm^{-1}.

N-(2-(*tert*-Butyl)-2*H*-tetrazol-5-yl)-1-phenyl-1*H*-tetrazol-5-amine (12a). Compound **11a** (0.045 g, 0.13 mmol) was refluxed in 2 mL of 10% HCl for 3 h. After cooling to room temperature the precipitate was filtered and recrystallized from ethyl alcohol to give **12a** as a white solid. Yield 0,017 g (56 %). Mp 133–135 °C. ^{1}H NMR (500.0 MHz, DMSO-d_6), δ: 11.06 (br, s, 1H, NH), 7.74–7.53 (m, 5H, Ph), 1.52 (s, 9H, t-Bu) ppm; ^{13}C NMR (125.7 MHz, DMSO-d_6), δ: 161.1 (CN_4), 151.9 (CN_4), 133.8 (C^4, Ph), 130.4 (C^1, Ph), 130.2 ($C^{2,6}$, Ph), 124.8 ($C^{3,5}$, Ph), 64.4 (<u>C</u>Me_3), 28.9 (3Me) ppm. Anal. Calcd for $C_{12}H_{15}N_9$: C, 50.5; H, 5.3; N, 44.2. Found: C, 50.7; H, 5.3; N, 44.6. IR (neat), ṽ: 2986 (w), 2909 (w), 2828 (w), 2770 (w), 1655 (w), 1615 (s), 1593 (m), 1549 (m), 1524 (s), 1497 (s), 1455 (m), 1402 (w), 1370 (m), 1344 (m), 1314 (m), 1287 (w), 1259 (w), 1237 (w), 1201 (m), 1188 (m), 1172 (w), 1157 (w), 1129 (m), 1093 (m), 1072 (m), 1046 (m), 1012 (s), 988 (w), 939 (w), 918 (w), 861 (m), 831 (m), 814 (w), 760 (s), 748 (s), 722 (s), 685 (s), 595 (m), 554 (m), 503 (w), 476 (w), 449 (w) cm^{-1}.

1-Methyl-*N*-(1-phenyl-1*H*-tetrazol-5-yl)-1*H*-tetrazol-5-amine (12b). Compound **11b** (0.1038 g, 0.35 mmol) was refluxed in 2.5 mL of 10% HCl for 6 h. After cooling to room temperature the precipitate was filtered and recrystallized from ethyl alcohol to give **12b** as a white solid. Yield 0,037 g (44 %). Mp 187–190 °C. ^{1}H NMR (500.0 MHz, DMSO-d_6), δ: 7.89–7.50 (m, 5H, Ph), 3.80 (s, 3H, Me) ppm; ^{13}C NMR (125.7 MHz, DMSO-d_6), δ: 153.2 (CN_4), 151.1 (CN_4), 134.4 (C^1, Ph), 130.0 (C^4, Ph), 129.4 ($C^{2,6}$, Ph), 123.3 ($C^{3,5}$, Ph), 32.9 (Me) ppm. Anal. Calcd for $C_9H_9N_9$: C, 44.4; H, 3.7; N, 51.8. Found: C, 44.3; H, 3.5; N, 51.7. IR (neat), ṽ: 3016 (m), 1671 (w), 1623 (s), 1608 (s), 1590 (s), 1537 (s), 1498 (s), 1491 (s), 1450 (s), 1420 (w), 1401 (s), 1328 (w), 1301 (w), 1282 (w), 1268 (w), 1256 (w), 1219 (m), 1175 (w), 1160 (w), 1141 (m), 1115 (s), 1198 (m), 1069 (m), 1023 (s), 1005 (m), 980 (m), 911 (w), 827 (m), 810 (m), 754 (s), 713 (s), 682 (s), 663 (s), 616 (w), 548 (w), 508 (m), 498 (m), 473 (w), 452 (s) cm^{-1}.

Results from calculations of UV–vis spectra of 8a

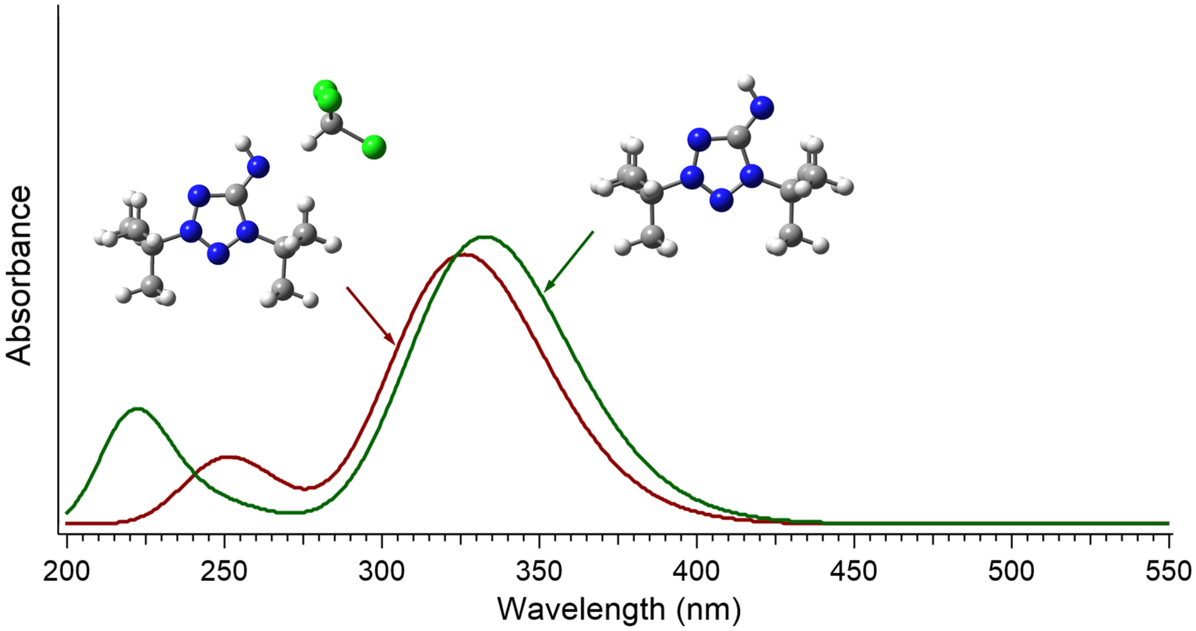

Figure S1. The TD-tHCTHhyb/6-311+G(2d,p) calculated absorption spectra of **8a** in chloroform for two different models: continuum SMD (green curve); combined continuum SMD and super-molecule model, taking into account the formation of a hydrogen bond between **8a** and chloroform (red curve).

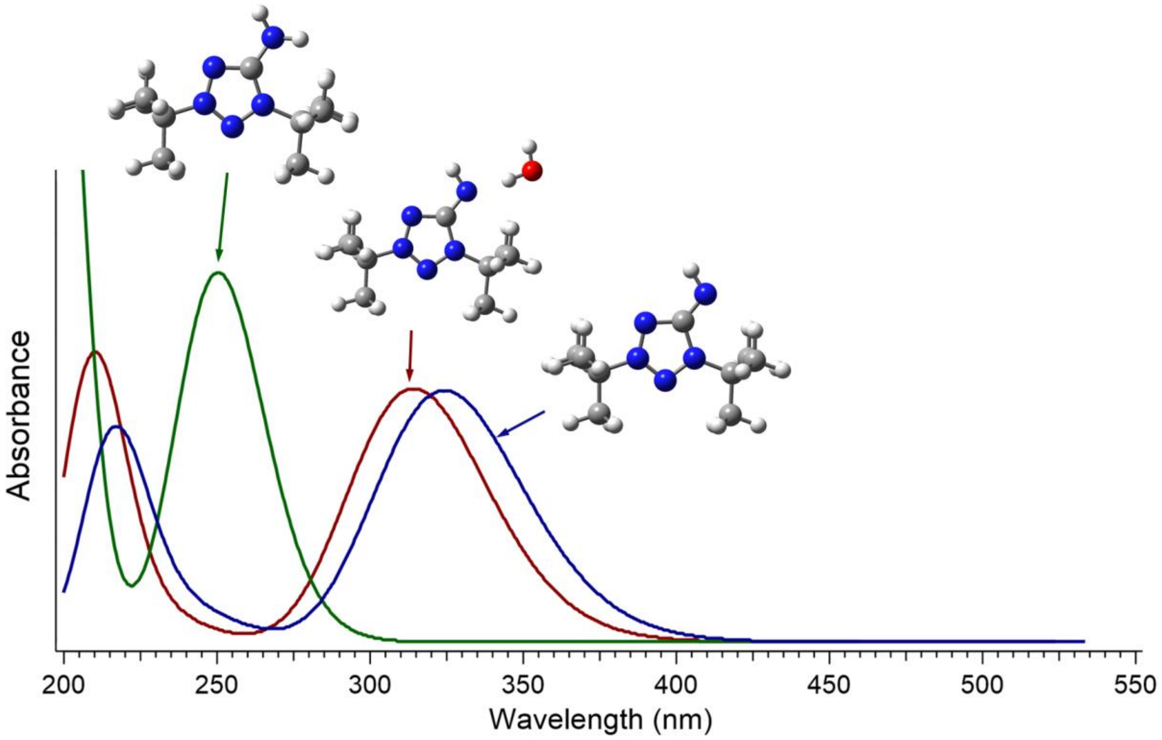

Figure S2. The TD-tHCTHhyb/6-311+G(2d,p) calculated absorption spectra of **8a** in water for three different models: continuum SMD (blue curve); combined continuum SMD and super-molecule model, taking into account the formation of a hydrogen bond between **8a** and chloroform (red curve); continuum SMD model, taking into account the protonation of **8a** in water solution (green curve).

TGA/DSC details for compounds 8a and 10

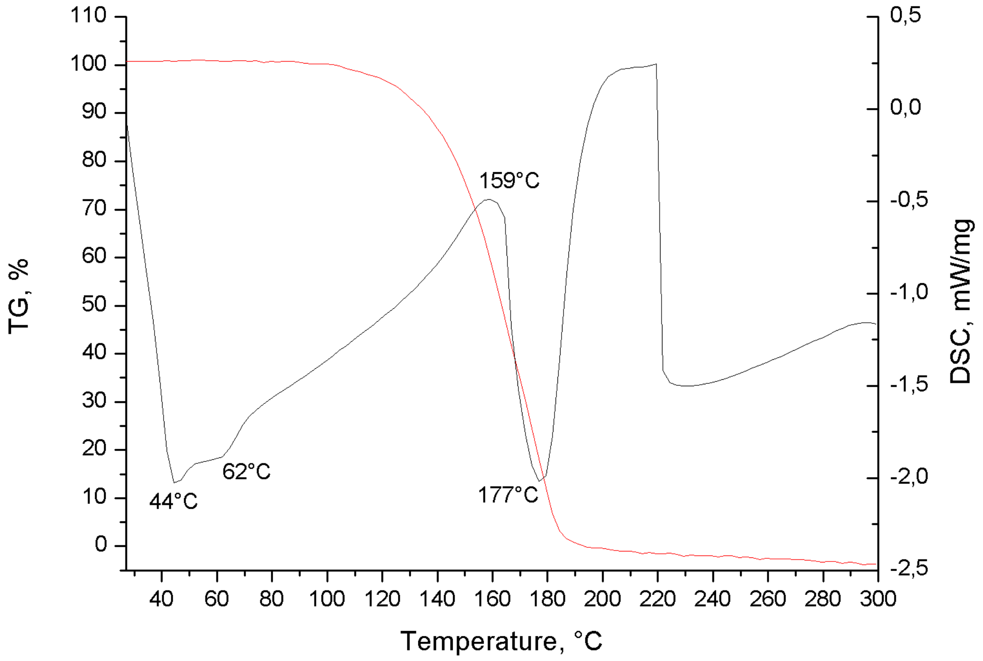

Figure S3. TG and DSC curves of compound **8a**

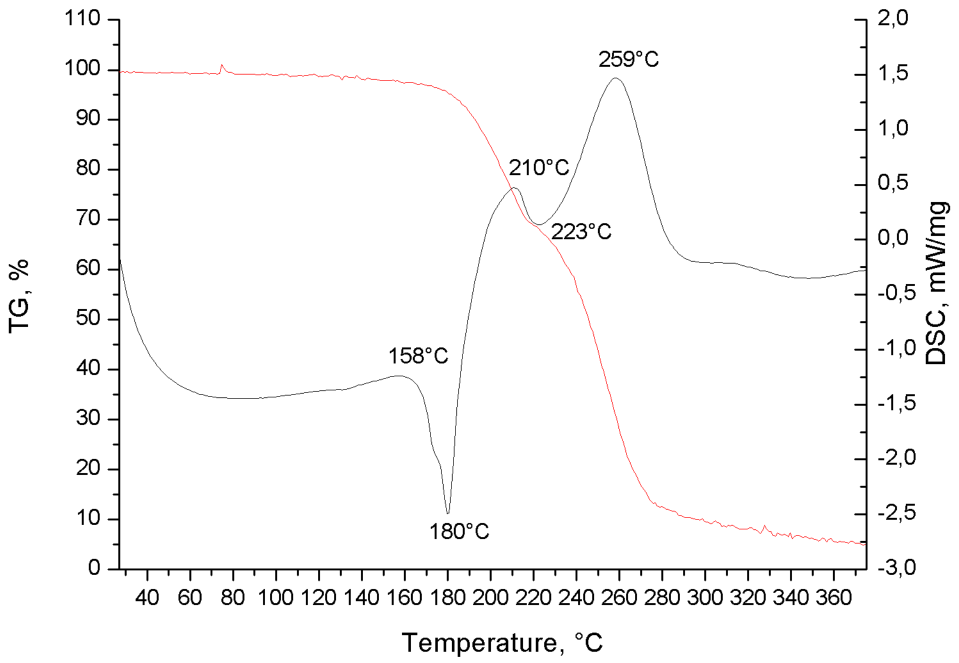

Figure S4. TG and DSC curves of bistetrazolium-5-aminide **10**

NMR spectra of the synthesized compounds

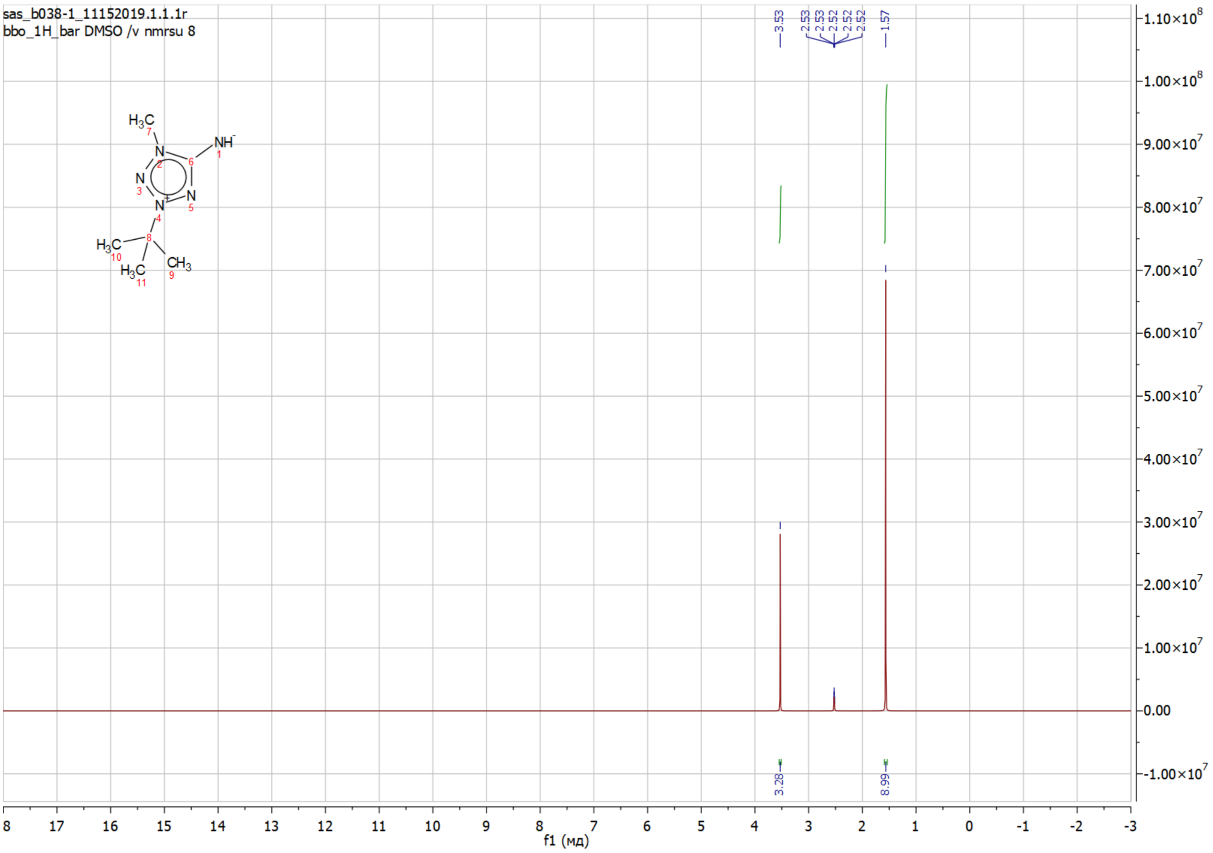

Figure S5. ^{1}H NMR of 1-methyl-3-*tert*-butyltetrazolium-5-aminide (**8b**)

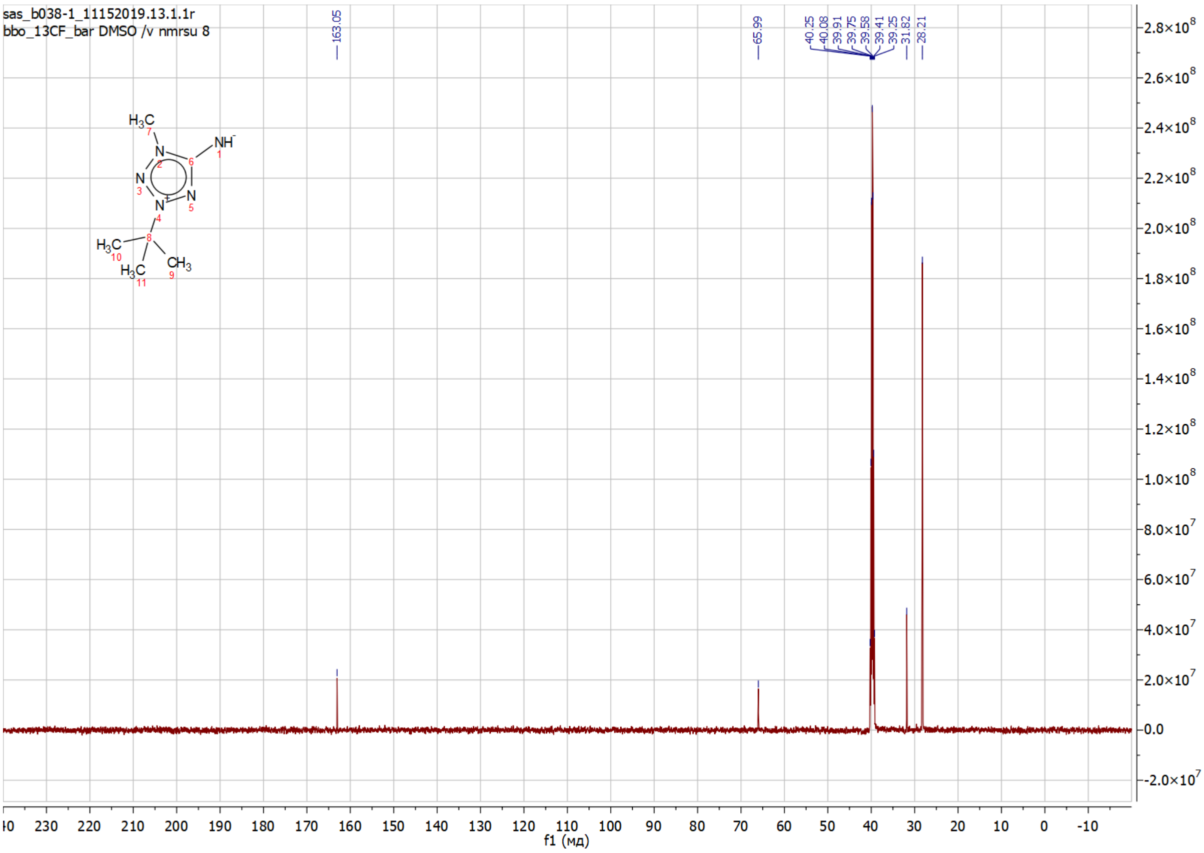

Figure S6. ^{13}C NMR of 1-methyl-3-*tert*-butyltetrazolium-5-aminide (**8b**)

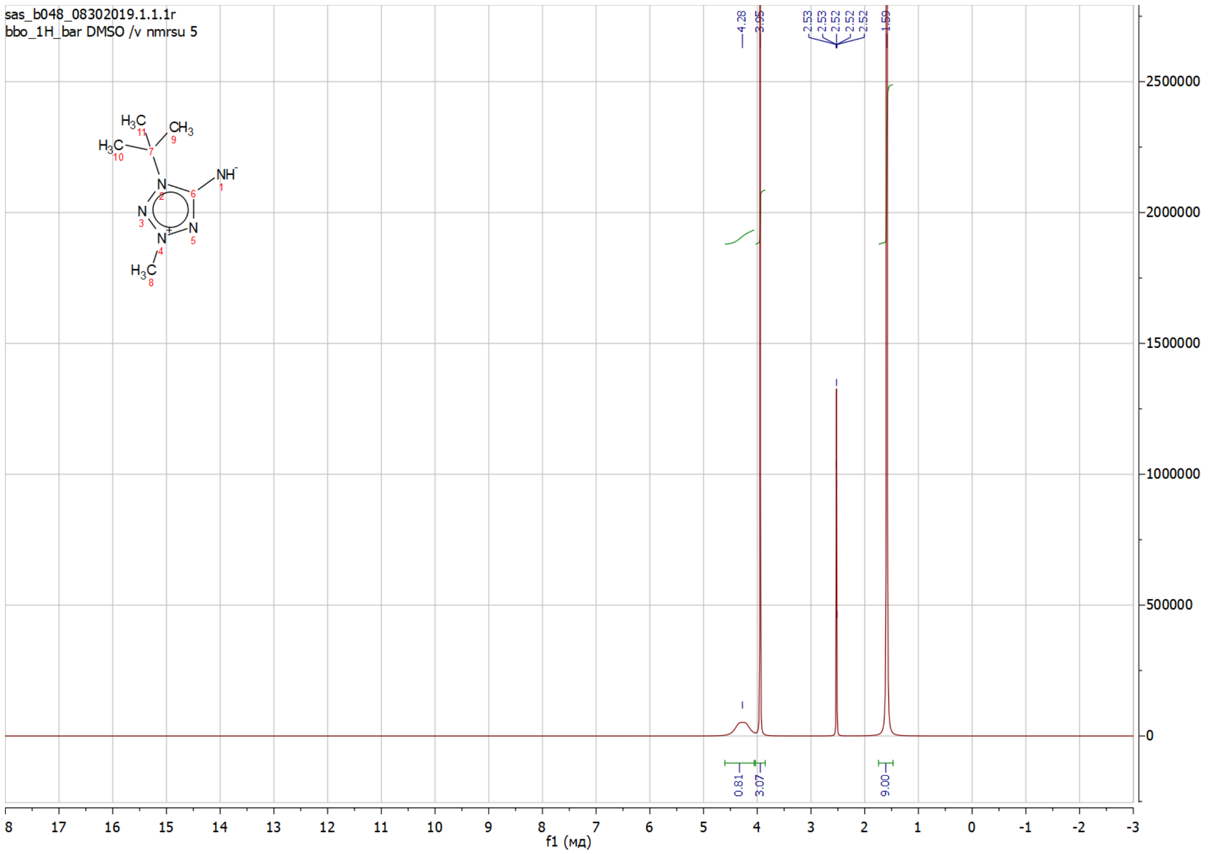

Figure S7. ^{1}H NMR of 3-methyl-1-*tert*-butyltetrazolium-5-aminide (**8c**)

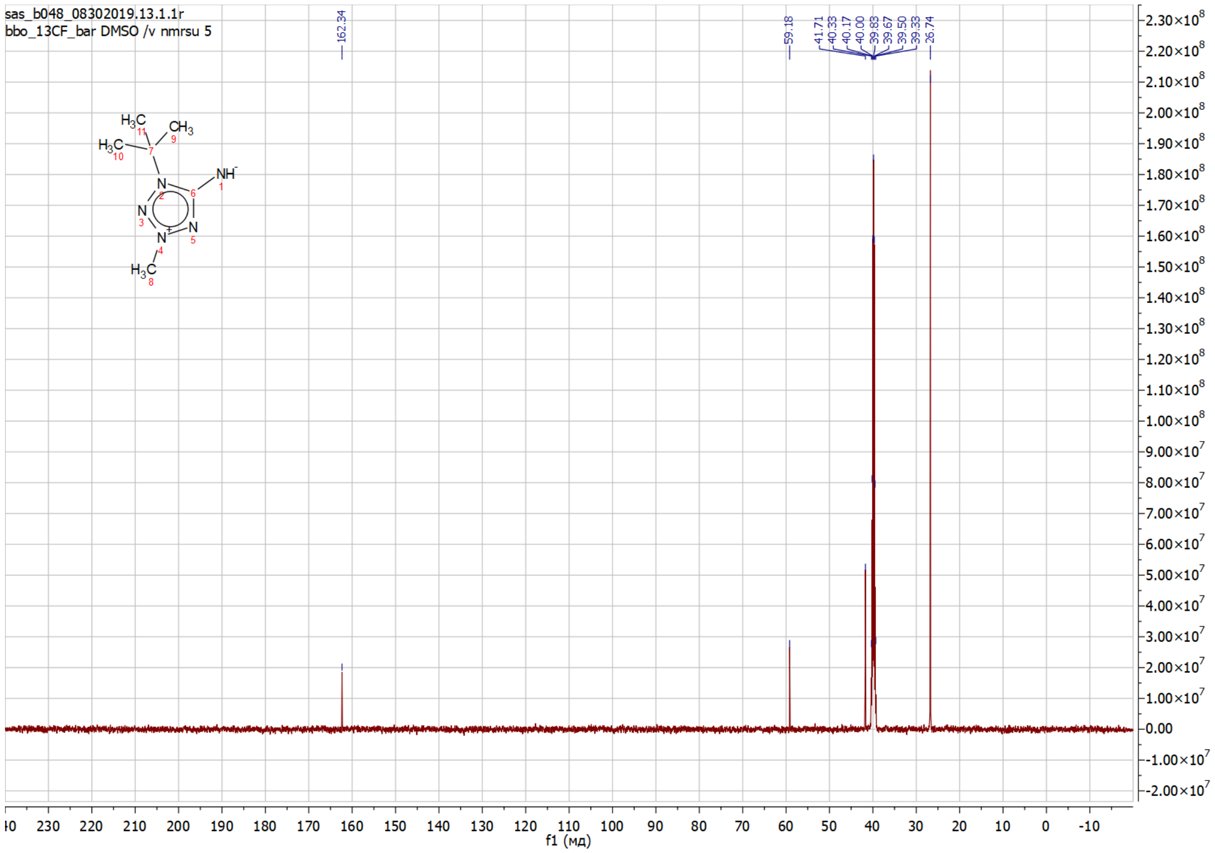

Figure S8. ^{13}C NMR of 3-methyl-1-*tert*-butyltetrazolium-5-aminide (**8c**)

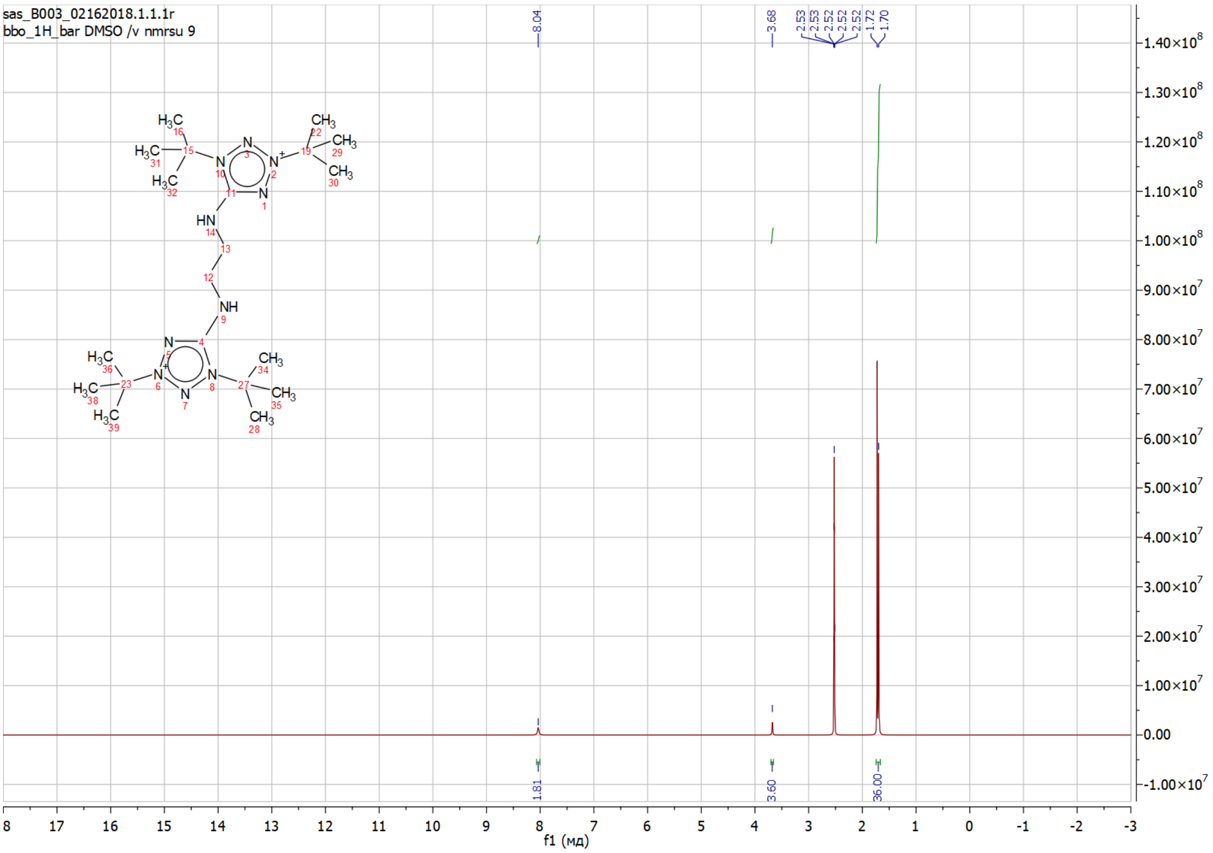

Figure S9. ^{1}H NMR of bistetrazolium salt **9**

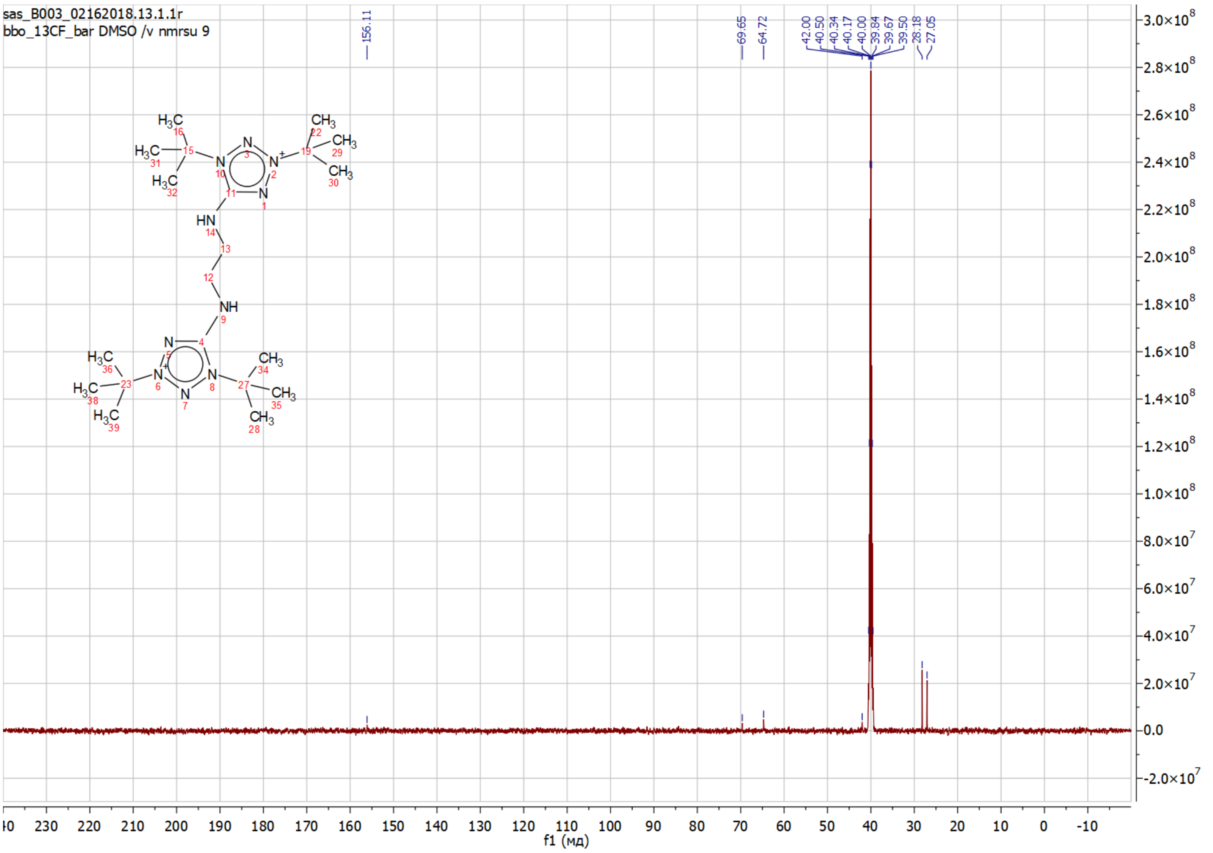

Figure S10. ^{13}C NMR of bistetrazolium salt **9**

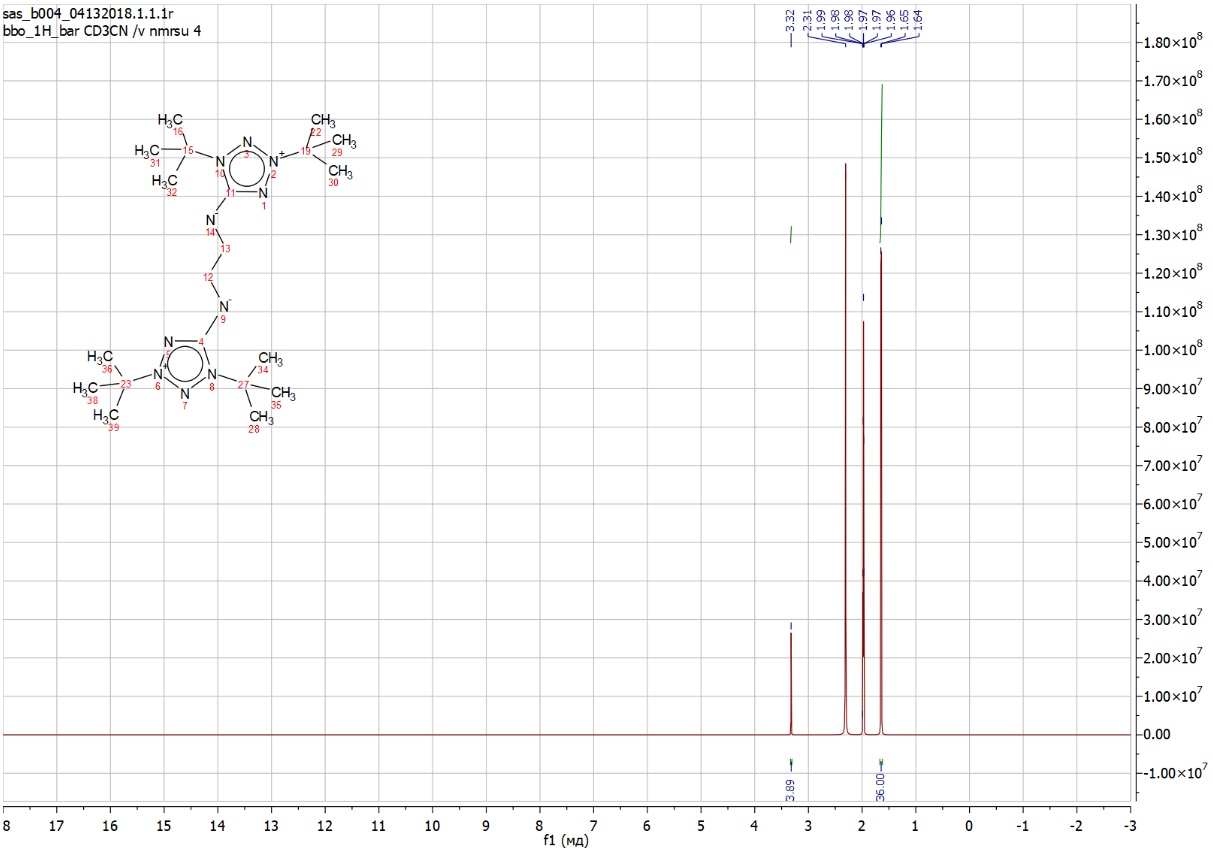

Figure S11. ^{1}H NMR of bistetrazolium-5-aminide **10**

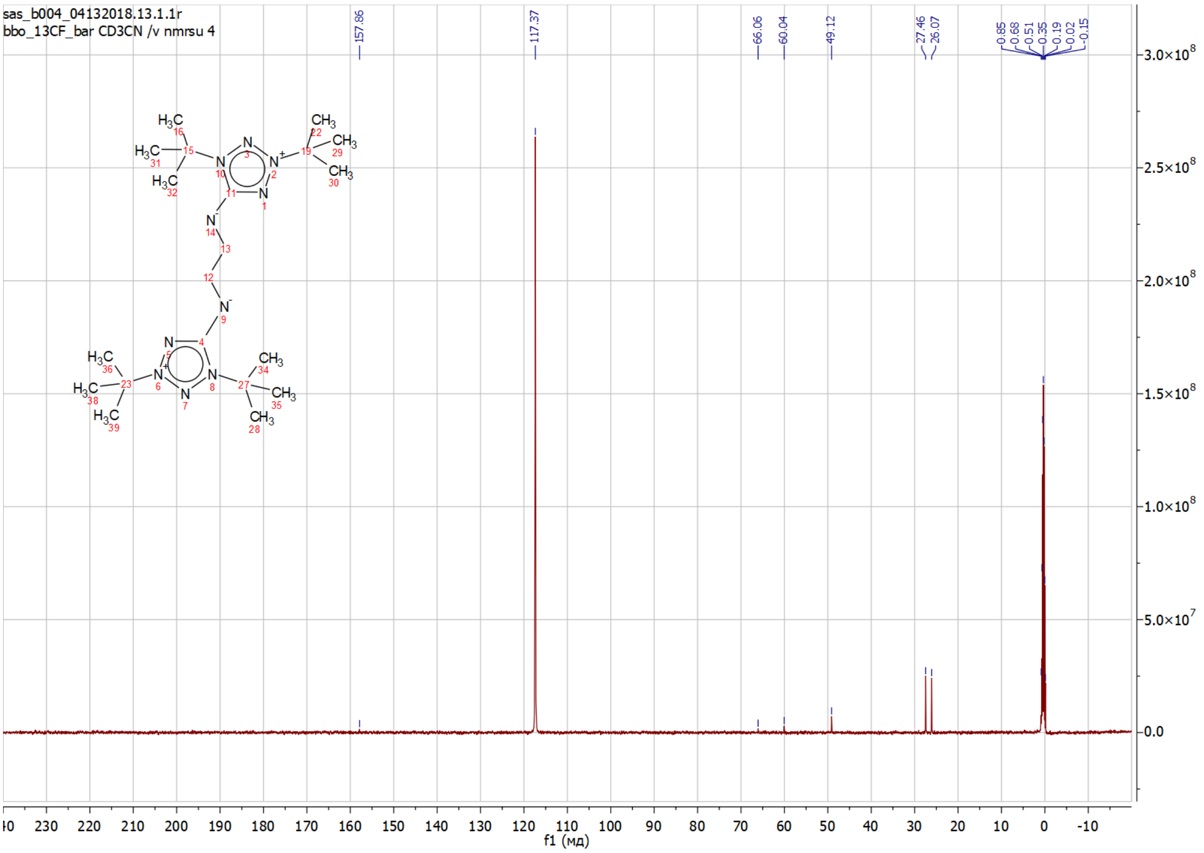

Figure S12. ^{13}C NMR of bistetrazolium-5-aminide **10**

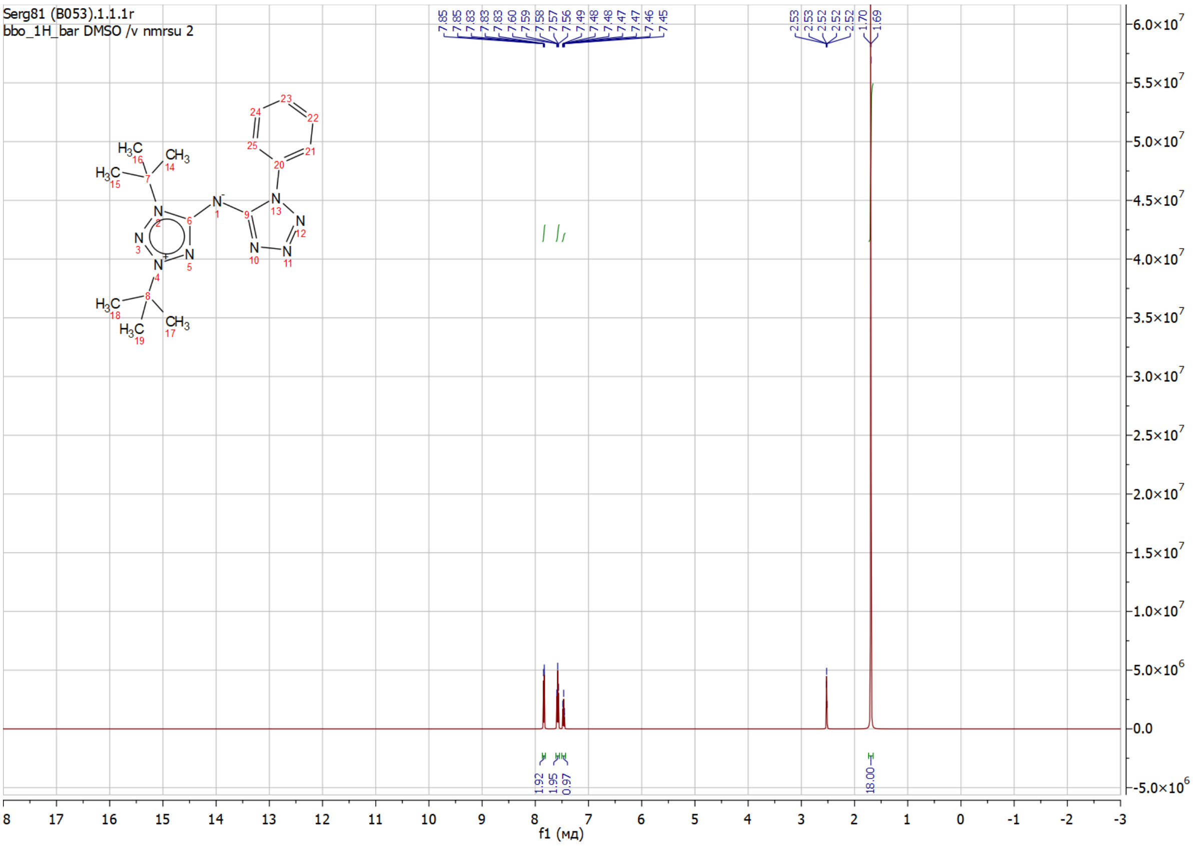

Figure S13. ^{1}H NMR of (1,3-di-*tert*-butyl-1*H*-tetrazol-3-ium-5-yl)(1-phenyl-1*H*-tetrazol-5-yl)amide (**11a**)

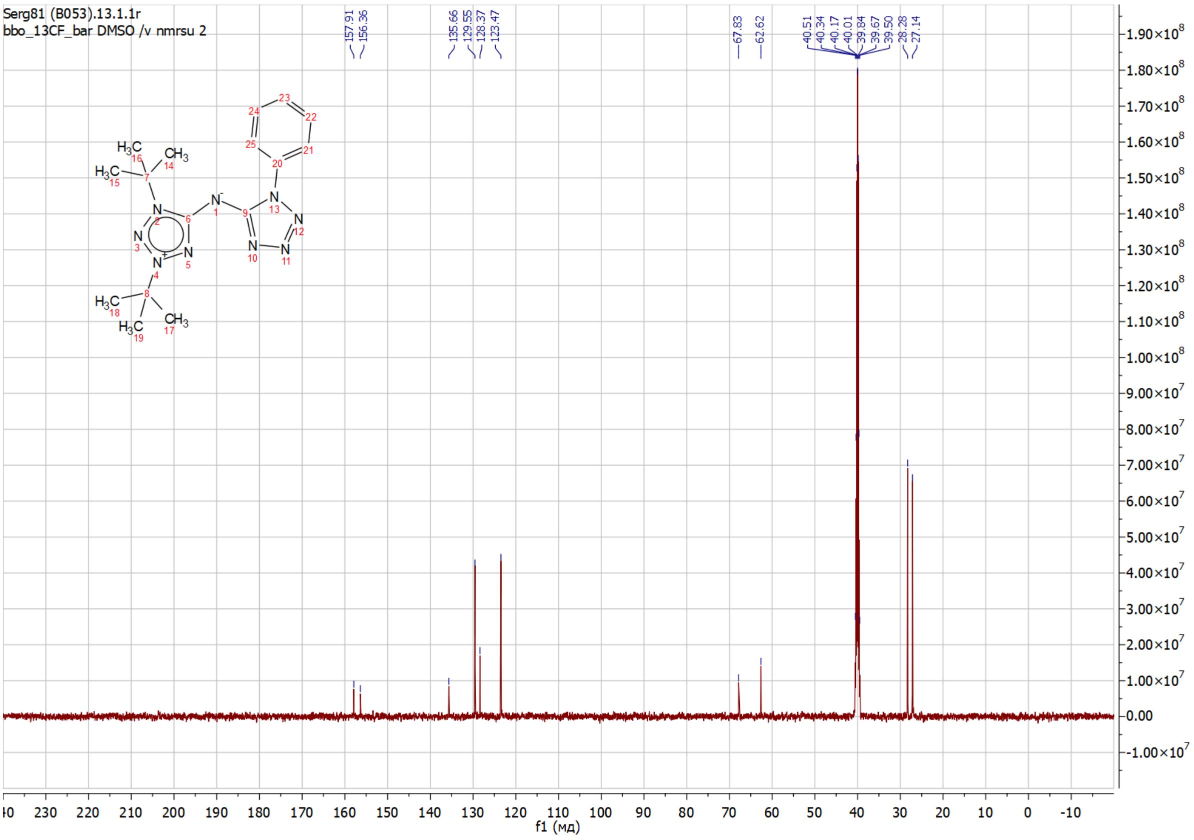

Figure S14. ^{13}C NMR of (1,3-di-*tert*-butyl-1*H*-tetrazol-3-ium-5-yl)(1-phenyl-1*H*-tetrazol-5-yl)amide (**11a**)

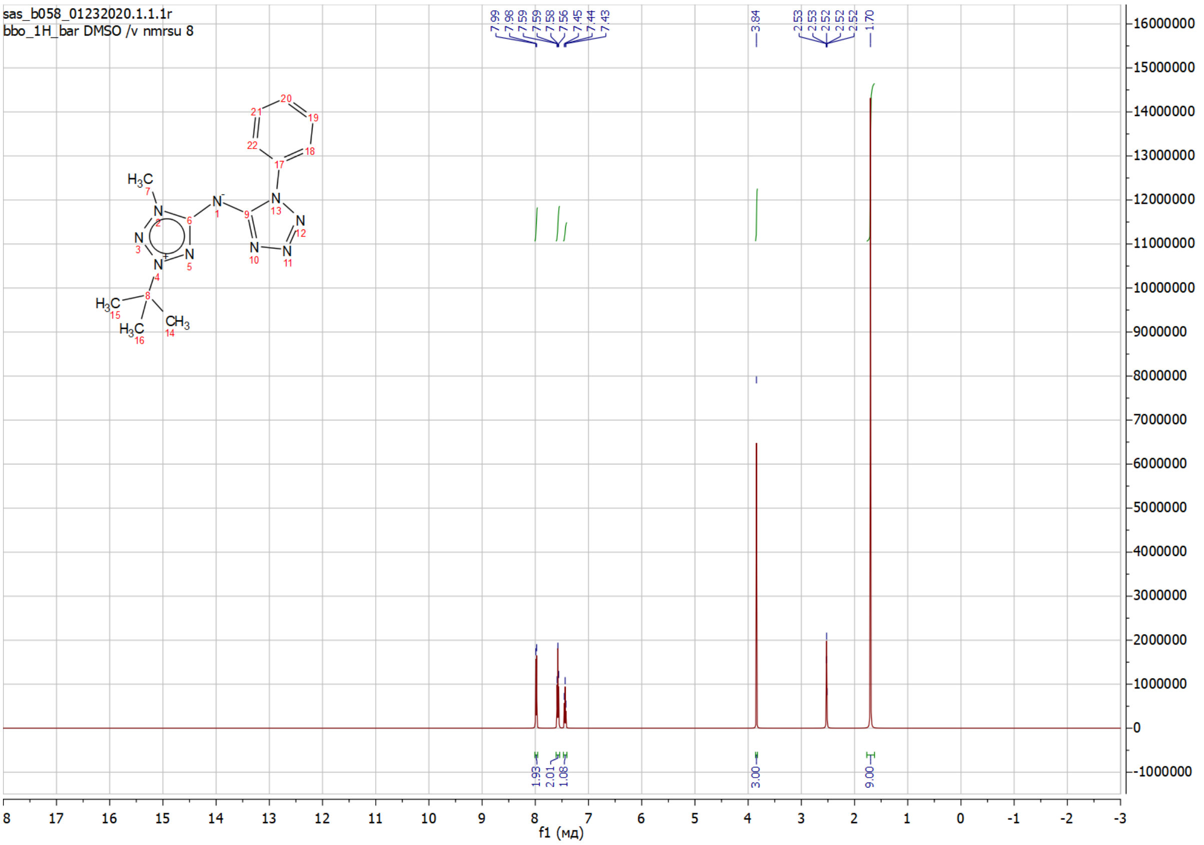

Figure S15. ^{1}H NMR of (1-methyl-3-*tert*-butyl-1*H*-tetrazol-3-ium-5-yl)(1-phenyl-1*H*-tetrazol-5-yl)amide (**11b**)

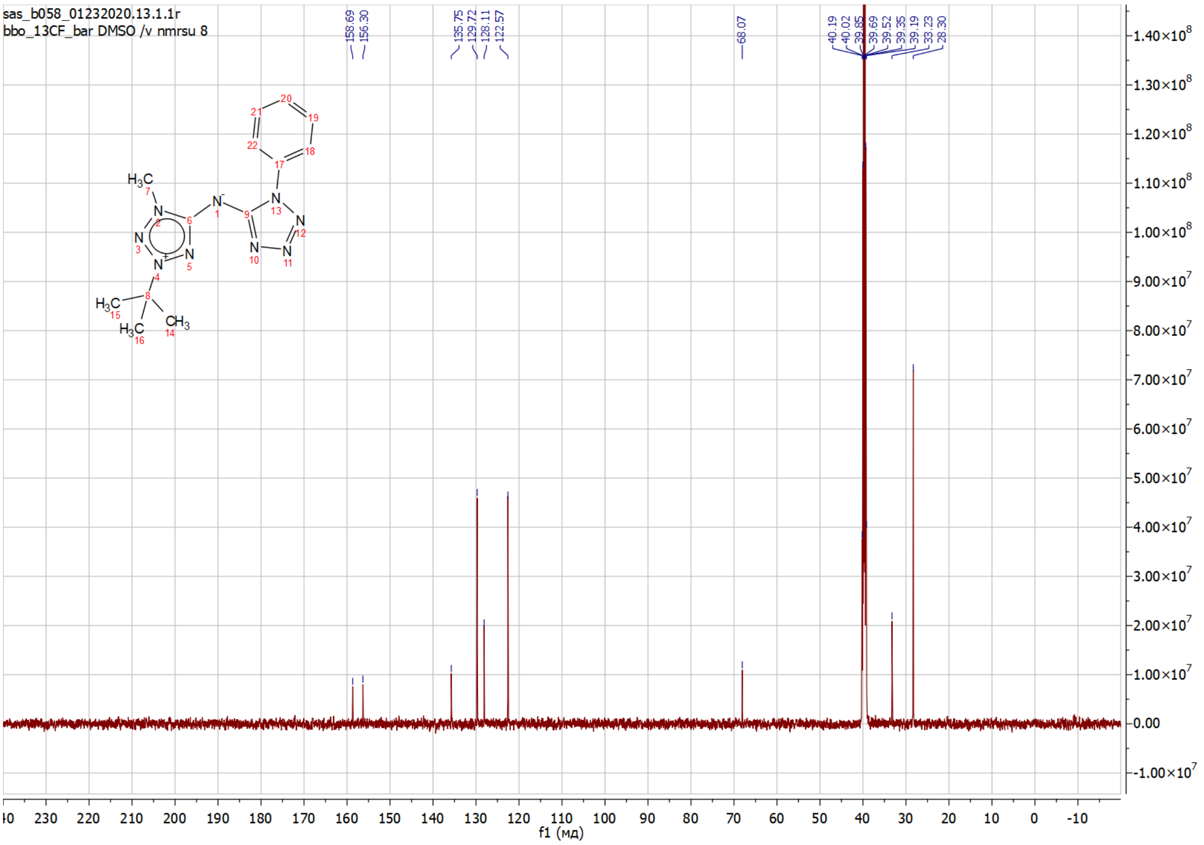

Figure S16. ^{13}C NMR of (1-methyl-3-*tert*-butyl-1*H*-tetrazol-3-ium-5-yl)(1-phenyl-1*H*-tetrazol-5-yl)amide (**11b**)

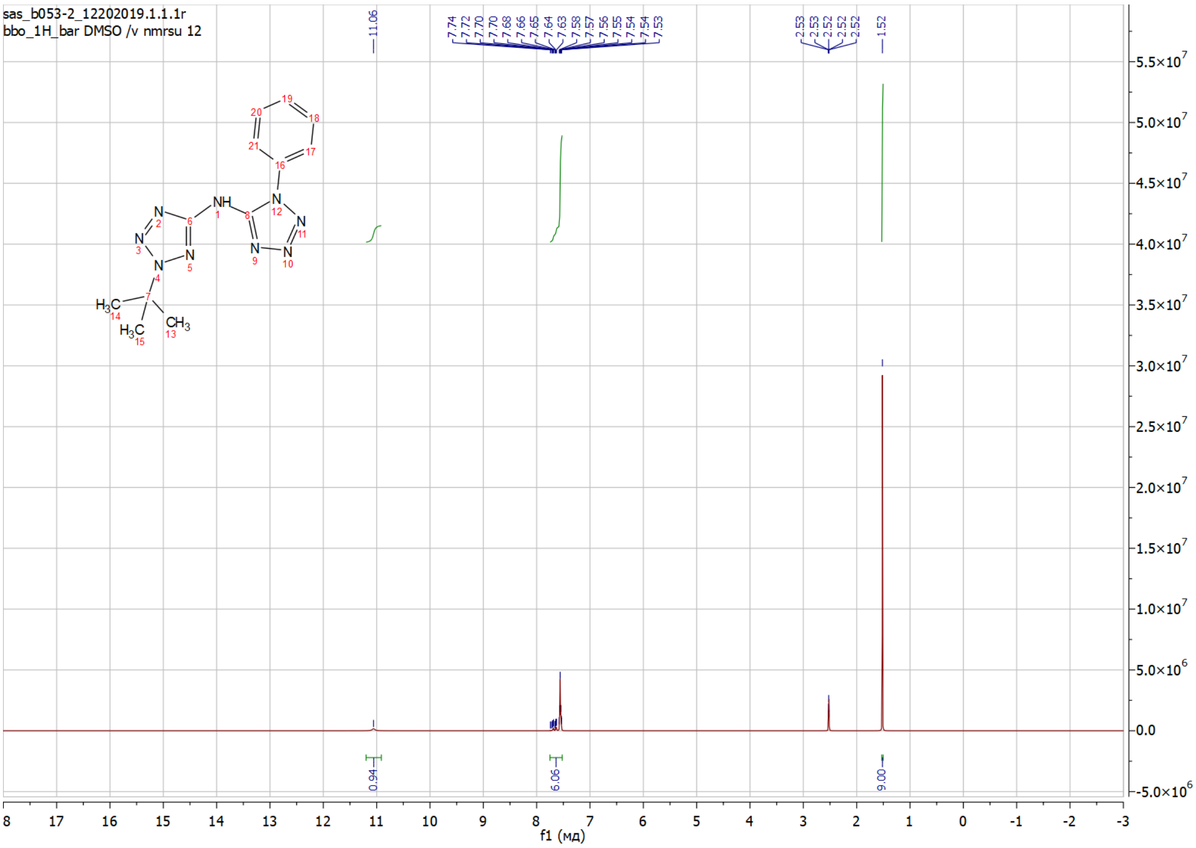

Figure S17. ^{1}H NMR of *N*-(2-(*tert*-butyl)-2*H*-tetrazol-5-yl)-1-phenyl-1*H*-tetrazol-5-amine (**12a**)

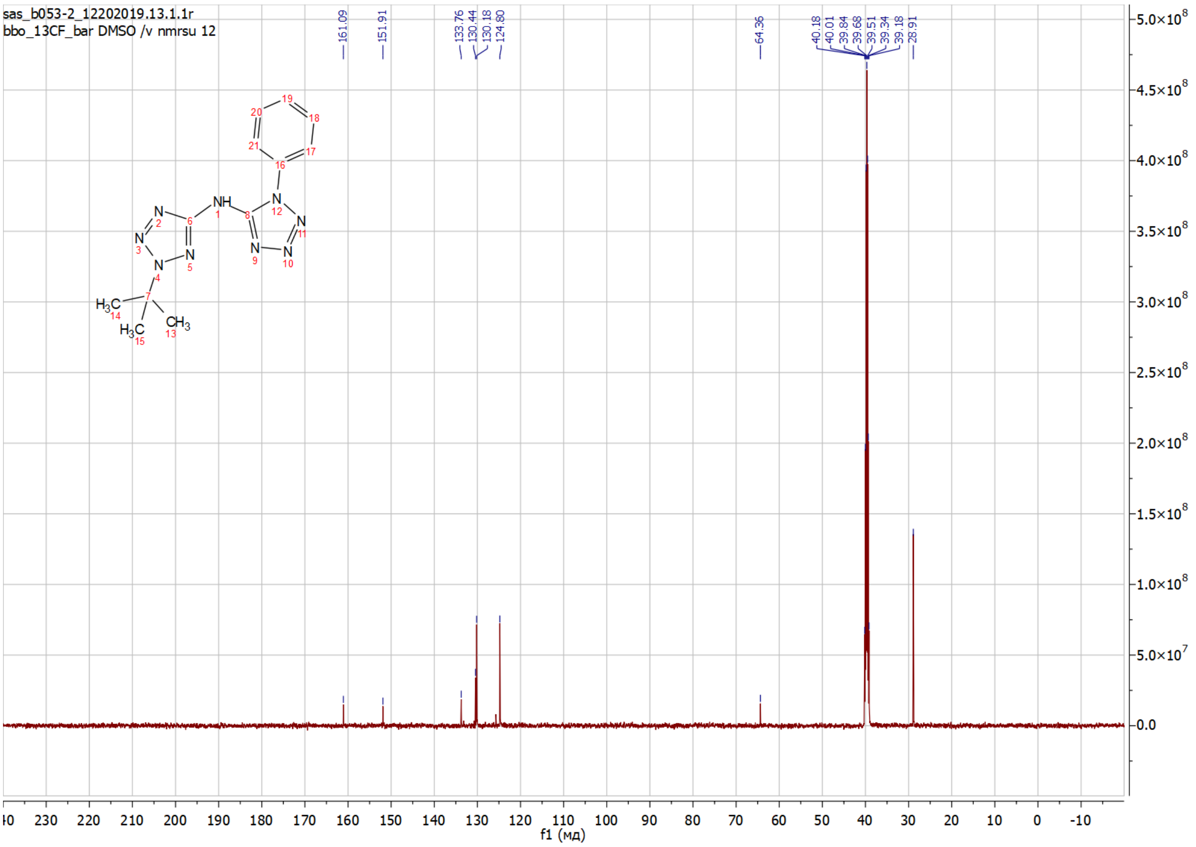

Figure S18. ^{13}C NMR of *N*-(2-(*tert*-butyl)-2*H*-tetrazol-5-yl)-1-phenyl-1*H*-tetrazol-5-amine (**12a**)

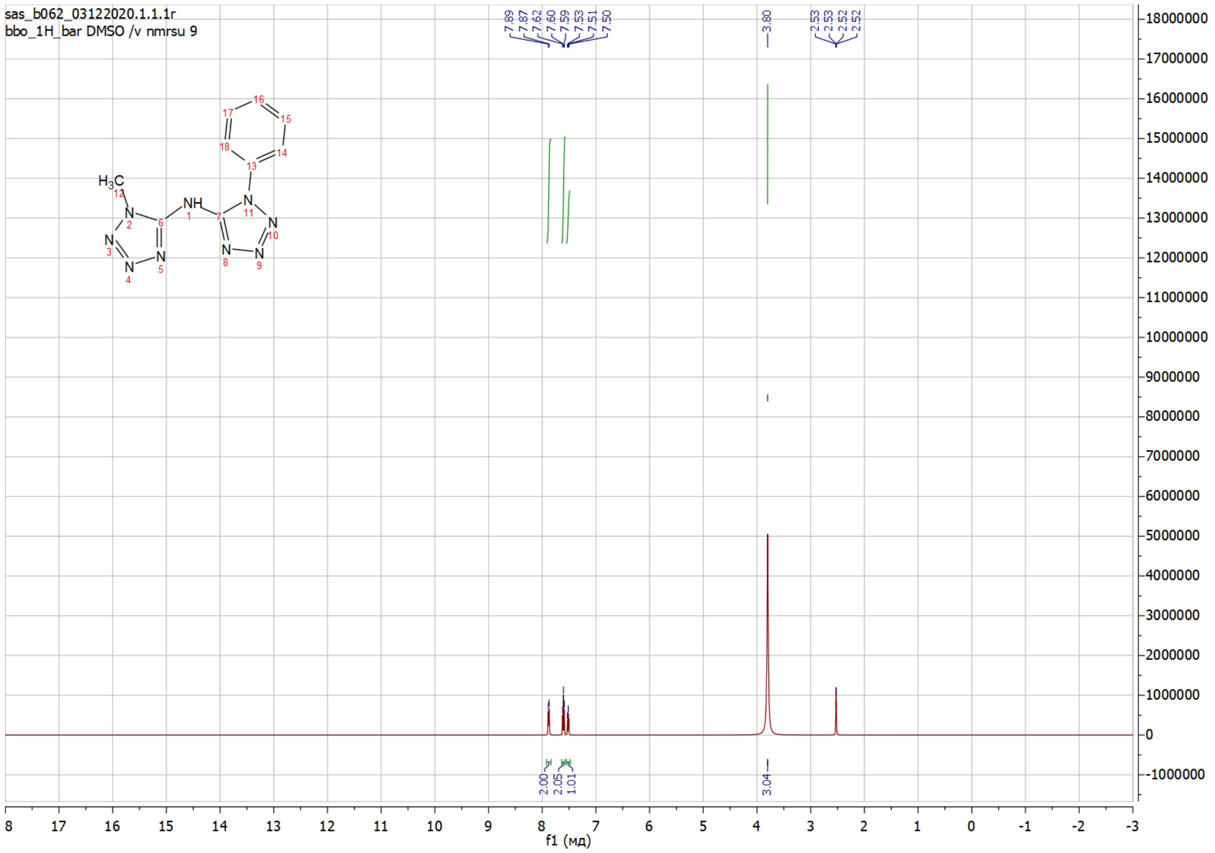

Figure S19. ^{1}H NMR of 1-methyl-*N*-(1-phenyl-1*H*-tetrazol-5-yl)-1*H*-tetrazol-5-amine (**12b**)

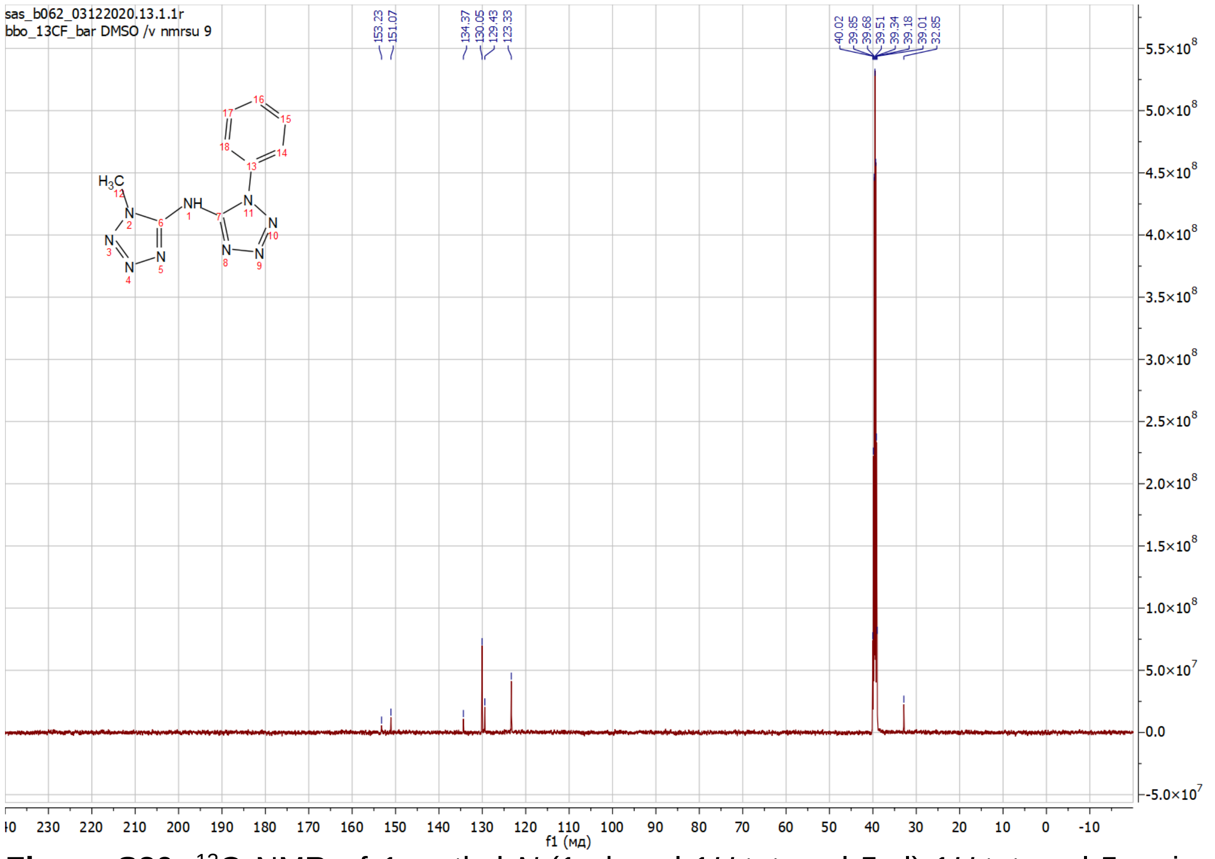

Figure S20. ^{13}C NMR of 1-methyl-*N*-(1-phenyl-1*H*-tetrazol-5-yl)-1*H*-tetrazol-5-amine (**12b**)
